# Inshore marine coastal zone migration patterns in Atlantic salmon post‐smolts emigrating from eight rivers in north‐east Scotland

**DOI:** 10.1111/jfb.70280

**Published:** 2025-11-20

**Authors:** Colin E. Adams, Ruaidhri Forrester, Matthew Newton, Angus J. Lothian, Hannele M. Honkanen, Davide Thambithurai, Lorna Wilkie, Melanie A. Smith, Marcus Walters, Richard Miller, Karen Muller, Brian Shaw, Steve Burns, Chris Conroy, Chris Daphne, Robert Laughton, Mirella Toth, Keith Williams, Sean Robertson, Ross Glover, Ben Seaman, Martyn C. Lucas, Louise Chavarie, Jessica R. Rodger

**Affiliations:** ^1^ Scottish Centre for Ecology and the Natural Environment, School of Biodiversity, One Health and Veterinary Medicine, University of Glasgow Glasgow UK; ^2^ Atlantic Salmon Trust, The Walled Garden, Kilgraston Perth UK; ^3^ Department of Biological Sciences University of Calgary Calgary Alberta Canada; ^4^ The Deveron, Bogie and Isla Rivers Charitable Trust Avochie UK; ^5^ The Ness District Salmon Fishery Board, Beauly House, Dochfour Business Centre Inverness UK; ^6^ The Spey Fisheries Board, Nether Borlum Cottage Aberlour UK; ^7^ The Findhorn, Nairn and Lossie Rivers Trust, Logie Steading Forres UK; ^8^ The Kyle of Sutherland Fisheries, Bank House Ardgay UK; ^9^ Cromarty Firth Fishery Board, Clark Thomson House, Fairways Business Park Inverness UK; ^10^ Department of Biosciences Durham University Durham UK; ^11^ Faculty of Environmental Sciences and Natural Resources Management, Norwegian University of Life Sciences Ås Norway

**Keywords:** migration, migration pathways, nearshore coastal, *Salmo salar*

## Abstract

Atlantic salmon, *Salmo salar*, migrate through multiple habitat types, each having the potential to impact differently upon migration success. The inshore marine coastal zone is arguably disproportionately impacted by potential stressors on populations. We investigated the migration of Atlantic salmon post‐smolts (*n* = 898) from eight rivers (populations) through the inshore marine coastal zone over a 2‐year period using acoustic telemetry. Migration success through marine inlets where fish first entered seawater, and through the wider inshore marine coastal area (the Moray Firth, north‐east Scotland) was consistently high across populations and across years (mean = 77%). There was no evidence of the high inshore marine predation rates reported elsewhere. Post‐smolts utilised well‐defined inshore marine migration pathways that were broadly consistent among populations and over time. The pace of migration through the inshore marine coastal habitat was also consistently rapid across populations and time, with the period of migration being seasonally constrained (mean across population = 36.5; range 18–45 days). Taken together with existing literature, this study suggests that the patterns of migration of Atlantic salmon post‐smolts through the inshore marine coastal zone are likely highly environment specific.

## 1 INTRODUCTION

Long‐distance migrations in animals are generally associated with elevated rates of mortality (Cresswell et al., 2011; Guillemain et al., 2010; Owen & Black, 1991; Sillett & Holmes, 2002; Strandberg et al., 2009). For Atlantic salmon, *Salmo salar* L, which typically migrate from their natal breeding grounds in rivers to feed in the northern North Atlantic (Friedland, 1998; Holm et al., 2000; Rikardsen et al., 2021; Thorstad et al., 2012), the risks associated with this migration appear to have increased over the past decade or more (Adams et al., 2022; Thorstad et al., 2021). Given that the numbers of adult Atlantic salmon returning from sea to breed are declining in most monitored populations across its range (Chaput et al., 2019; Dadswell, 2000; Kocik & Brown, 2002; Mills et al., 2013; Potter & Crozier, 2000), there is a pressing need to understand the processes involved in migration of this species.

Many of the pressures that may influence migration success in Atlantic salmon are either directly or indirectly linked to human development activities (Lennox et al., 2021; Thorstad et al., 2021). Several studies have reviewed the pressures that are likely impacting salmon populations during migration (Forseth et al., 2017; Gillson et al., 2022; Parrish et al., 1998; Scottish Government, 2022; Thorstad et al., 2012). Gillson et al. (2022), for example, provide a comprehensive review of the main marine pressures on Atlantic salmon. These include a range of activities disproportionately found in inshore marine coastal zones (compared to riverine and offshore marine environments), which are exposed to relatively intense human activity. Such activities include salmon aquaculture, offshore renewable energy generation, electromagnetic field production (a by‐product of underwater power cables), tidal barrage development, exploitation in fisheries and incidental by‐catch. Thus, it is likely that the inshore coastal marine phase of migration presents a particularly vulnerable period for Atlantic salmon, with much of the associated risk stemming from human activities that could potentially be mitigated through effective management.

In contrast to the riverine component of salmon migration, which is relatively well studied, marine migration is much less well understood (Adams et al., 2022; Gilbey et al., 2021; Thorstad et al., 2011). This is largely because of the very substantive logistical resource requirements involved in executing such studies at sea (Lennox et al., 2023). Much of what we do know of the marine migration of Atlantic salmon comes from opportunistic and targeted offshore trawling studies and genetic and microtracer studies (Gilbey et al., 2021). These have shown that Atlantic salmon originating from rivers across the southern part of the species' range in Europe migrate to, and disperse over, a relatively broad area of the Norwegian Sea to feed (Gilbey et al., 2021) and are most abundant around the relatively shallow areas of the Vøring Plateau (Holm et al., 2000; Holst, 2011). Atlantic salmon originating from European rivers, as well as those from North American rivers, are also known to feed in the Labrador Sea in the western North Atlantic, most notably to the west of Greenland (Hansen & Quinn, 1998; Reddin & Friedland, 1999) but also on the Grand Banks to the south‐east of Newfoundland (Reddin, 1985; Rikardsen & Dempson, 2011). In addition, targeted experimental trawl netting studies have captured juvenile post‐smolts originating from UK and Irish rivers along the continental shelf edge to the west of the UK and Ireland, suggesting this as a pathway for, at least some, populations heading for the Vøring Plateau (Gilbey et al., 2021; Holm et al., 2000).

Much less is known about the migration of Atlantic salmon during their passage through the coastal zones closest to shore, despite that it is this component of the migration where much of the mortality is thought to occur (Hansen & Quinn, 1998; MacLean et al., 2000; Potter & Crozier, 2000), and that this is where many of the human‐induced pressures are likely to occur (Forseth et al., 2017; Gillson et al., 2022; Scottish Government, 2022; Thorstad et al., 2012). One source of mortality in inshore marine waters is from predation, and several studies have suggested that elevated mortality, including from predation, may occur within coastal estuary and fjord habitats (Dieperink et al., 2001, 2002; Hedger et al., 2011; Vollset et al., 2025).

Modelling at‐sea Atlantic salmon migration behaviour has provided some valuable insights (e.g., Borland et al., 2025; Ohashi & Sheng, 2018), but empirical data are urgently needed to populate and validate these models. Studies examining Atlantic salmon migration across multiple rivers in the same years have revealed broadly synchronous timing of smolt and post‐smolt movements, although with some variation between rivers and similar inshore marine migration routes within but not among populations from different rivers (Lilly et al., 2024; Rodger, Honkanen, et al., 2025; Rodger, Lilly, et al., 2025). However, the pace of migration of Atlantic salmon smolts during the early part of their marine migration has been shown to vary with river of origin (Rodger, Lilly, et al., 2025).

In this study, we use acoustic telemetry to investigate the migration patterns and behaviour of post‐smolts through nearshore marine waters. We address the following three working hypotheses:

1. Migration success of post‐smolts in the nearshore coastal zone will be low.
2. The pathway utilised by post‐smolts in the nearshore coastal zone is river specific.
3. The timing of the marine migration of post‐smolts through the coastal zone will be broadly synchronous among rivers and over time.

In addition, we characterise a number of other elements of the migration through nearshore marine waters.

## 2 METHODS

### 2.1 Study area

**FIGURE 1:**
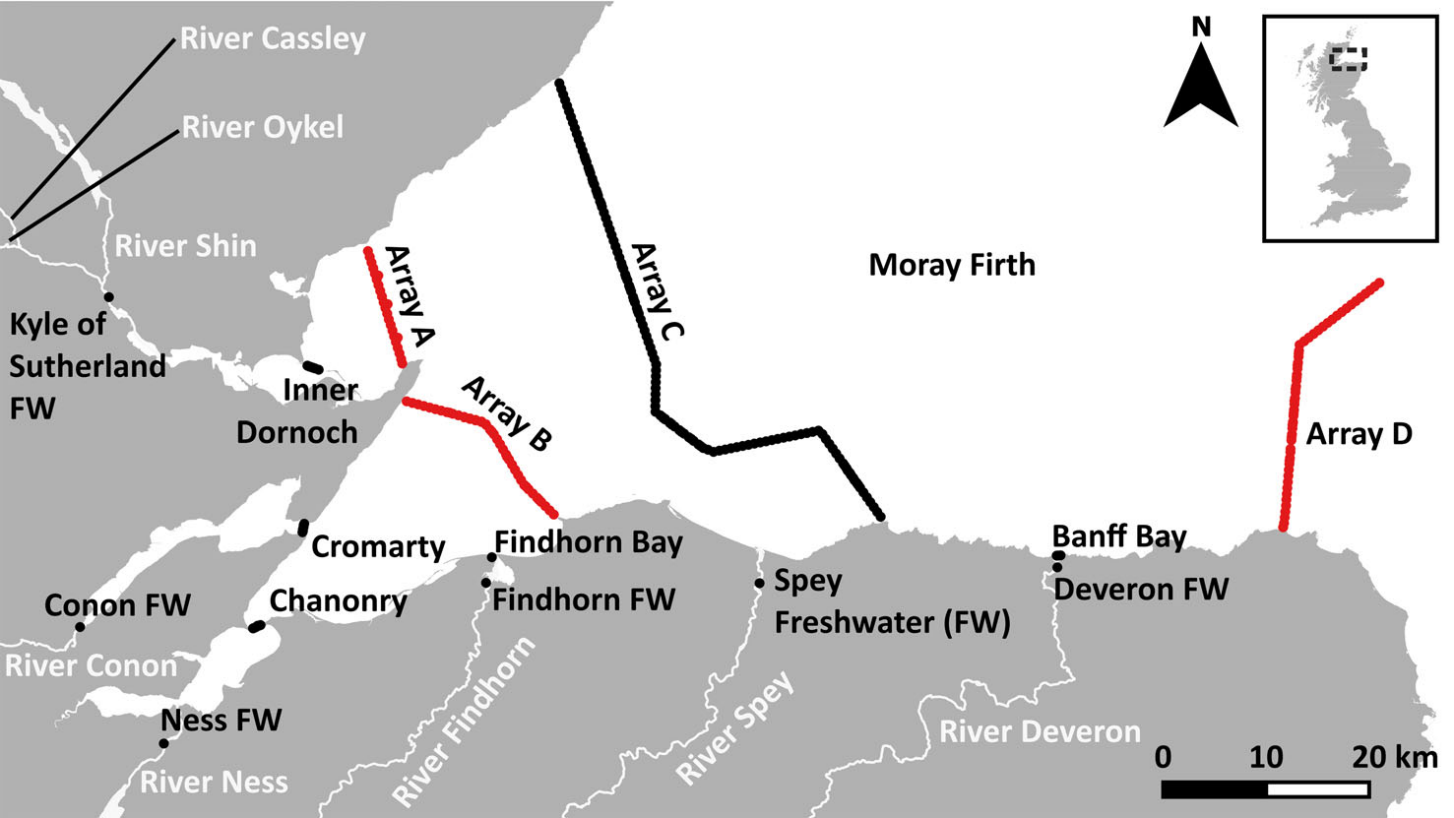
The Moray Firth study area. Study rivers are identified (white text). Individual receivers and receiver arrays deployed in both study years are indicated by a single or sequence of black dots. Red dots represent receivers deployed in 2019 only.

The Moray Firth (N 57° 47′ W 003° 38′) is a large coastal marine inlet of the North Sea on the north‐east coast of Scotland comprising an area of inshore marine coastal waters of around 145 km (east–west) to 70 km (north–south) (Figure 1). In this study, we investigate the migration of Atlantic salmon post‐smolts, originating from eight rivers that drain into the Moray Firth: the Deveron, Spey, Findhorn, Ness, Conon, Oykel, Cassley and Shin (Figure 1). The study period comprised the smolt and post‐smolt migration windows (March to August) in 2019 and 2021.

### 2.2 Receiver deployment

Acoustic receivers (Innovasea model VR2Tx and VR2‐AR) were deployed to detect smolts and post‐smolts entering/exiting three habitat type areas along their presumptive migration route through the Moray Firth. These habitat types were (a) the freshwater (natal) river, (b) the marine inlets at the mouth of each river (except the River Spey, which does not have an inlet) and (c) the inshore marine coastal zone (the Moray Firth) (Figure 1; Table S1).

Receivers were deployed at, or near, the mouth of each of the eight rivers in which fish were tagged [marked as freshwater (FW)] in Figure 1, see Table S1 and within 500 m downstream of the release site in both 2019 and 2021. The rivers Shin, Cassley and Oykel converge to form the (freshwater) Kyle of Sutherland immediately before discharging into marine waters (the Dornoch Firth); therefore, a single receiver was deployed in the Kyle of Sutherland (Figure 1) to determine marine entry for tagged smolts from these three rivers (Table S1). The location of the freshwater receivers remained consistent between years except for that of the Ness and the Conon freshwater receiver locations, which, for logistic reasons, differed by 150 and 210 m, respectively, between years (Figure 1). This movement had no material effect on the detection of smolts in the river, as the detection range exceeded the width of the channel at this point.

A total of five arrays were deployed in marine inlets (indicated as Inner Dornoch, Cromarty, Chanonry, Findhorn Bay and Banff Bay arrays; Figure 1) and four in inshore coastal marine waters (arrays A, B, C and D; Figure 1; Table S1). However, the number of arrays and receivers deployed in 2019 and 2021 differed. In 2019, a total of 278 receivers were deployed in all five marine inlets and four coastal arrays. In 2021, a total of 161 receivers were deployed across all five marine inlets, but the number of receivers in the Chanonry and Cromarty arrays was reduced from seven to six. Only one coastal marine array (Array C) was deployed in 2021 (Figure 1).

Receivers in the inshore coastal marine arrays (arrays A–D) were spaced at a mean interval of 603 m, [standard deviation (SD) = 167.5]. Deployment of sentinel tags identical to those used for tagging in this study showed that 50% of all pings emitted were detected at a mean range of 293 to 354 m among receivers on the principal array (Array C). In both years, receivers were deployed in March and recovered in August, by which time the expected battery life of the tags had passed, and tagged fish were expected to have migrated out of the study area. All receivers were deployed subsurface and without surface markers to avoid collision with passing craft.

### 2.3 Smolt capture and tagging

Across both years of the study, Atlantic salmon smolts (*n* = 1559) were captured using rotary screw traps deployed across all rivers (Figure 1). In 2021, smolts were tagged in all eight study rivers, whereas in 2019, smolts were not tagged in the River Cassley; thus only seven rivers were included in the study that year. Only fish with fork length greater than 128 mm, a body mass greater than 18 g and undergoing smoltification were selected for tagging (Eek and Bohlin, 2005; Sloat and Reeves, 2014). Fish were anaesthetised in a solution bath comprising river water and tricaine methanesulphonate (MS‐222; 100 mg L^−1^ buffered with sodium bicarbonate 100 mg L^−1^). Once fully anesthetised (identified as loss of equilibrium, slight operculum movement and no response to touch), fish were placed on a V‐shaped sponge saturated with river water and an incision of ~10 mm was made along the ventral abdominal wall, anterior to the pelvic girdle. A 69‐kHz coded acoustic transmitter (hereafter tag; Innovasea V7‐2L, 1.5 g in air, 7 mm diameter, 19.5 mm length) (identical tag and delay used at all sites) coded for a 30‐s delay (variable within the range 18 to 35 s) was inserted into the body cavity, and the incision was closed with two independent sutures secured with modified surgeon's knots. Tagged fish were allowed to recover in a tank filled with aerated river water. Once upright and swimming normally, fish were transferred to an in‐river holding cage, through which river water flowed and were released at the capture site after 1 h. All fish were released during daylight hours, the timing being defined by the tagging time but in most cased between 11 AM and 4 PM. In total, 798 smolts were tagged in 2019 and 761 smolts in 2021. The mean tag burden [weight of tags as a proportion of fish weight (in air)] was 6.0% (SD = 0.85; range 2.3–8.1). The mean number of fish tagged in each river in each year was 103.9 (range: 48–150; Table 1). A recent study has identified that different taggers may have different effects on survival and migration in salmon (Heim et al., 2024). Given the broad geographic scale over which this study was conducted and that fish needed to be tagged simultaneously in all rivers, multiple surgeons (*N* = 17) were used over the 2 years of the study. To mitigate a potential tagger effect, all taggers were trained together, all received regular revision training, all operated to the same standard operating procedure and one of the most‐qualified taggers made quality assurance visits to each tagging station at least once per week throughout the study. Fish were deemed to be continuing migration successfully, following tagging, if they were detected on a receiver located in the river within 500 m downstream of the release site.

**TABLE 1:** The latitude and longitude of the fish release site and year‐specific descriptive data for fish in this study for each river.

River	Latitude; longitude	Year	N tagged	Tagging date range (mm‐dd)	Mean fork length ± SD (mm)	Mean mass ± SD (g)	% successful river migration	Successful inlet migration *N* (%)	Successful Moray firth *N3* (%)
Shin	57° 56.8′ N; 4° 24.3′ W	2019	100	04–11 to 04–27	136.2 ± 4.83	24.2 ± 2.50	92%	68 (76%)	52 (77%)
		2021	100	04–16 to 04–25	140.2 ± 6.63	25.7 ± 3.60	82%	50 (76%)	40 (80%)
Cassley	57° 58.9′ N; 4° 35.2′ W	2021	48	04–25 to 05–04	140.1 ± 6.26	25.2 ± 3.91	68%	24 (80%)	18 (75%)
Oykel	57° 58.5′ N; 4° 38.2′ W	2019	149	04–11 to 05–03	137.0 ± 7.24	25.3 ± 3.94	70%	67 (69%)	48 (72%)
		2021	100	04–16 to 04–27	140.0 ± 7.47	25.7 ± 4.28	84%	66 (84%)	43 (65%)
Conon	57°33.2′ N; 4° 30.4′ W	2019	99	04–14 to 05–07	139.4 ± 5.39	26.6 ± 3.35	90%	33 (72%)	18 (54%)
		2021	100	04–16 to 05–07	145.4 ± 8.56	30.0 ± 5.53	65%	47 (75%)	37 (79%)
Ness	57° 29.0′ N; 4° 14.0′ W	2019	100	04–12 to 04–26	140.3 ± 10.91	28.8 ± 6.73	10%	8 (89%)	8 (100%)
		2021	120	04–06 to 04–30	139.82 ± 7.88	28.2 ± 5.10	20%	17 (79%)	12 (71%)
Findhorn	57° 37.8′ N; 3° 37.9′ W	2019	100	04–13 to 05–02	135.5 ± 6.05	23.7 ± 3.58	44%	54 (83%)	38 (70%)
		2021	93	04–17 to 05–23	137.19 ± 6.62	23.7 ± 3.16	86%	55 (67%)	34 (62%)
Spey	57° 37.3′ N; 3° 06.3′ W	2019	150	04–13 to 05–02	134.5 ± 3.59	24.0 ± 2.46	61%	NA	56 (37%)
		2021	100	04–14 to 05–02	135.5 ± 4.10	24.3 ± 2.37	41%	NA	34 (65%)
Deveron	57° 39.1′ N; 2° 31.5′ W	2019	100	04–13 to 04–30	133.6 ± 5.04	23.5 ± 2.66	51%	33 (87%)	NA
		2021	100	04–13 to 04–30	137.1 ± 4.83	25.1 ± 2.90	74%	47 (64%)	NA

*Note*: Fork length (mm) and mass (g) were measured at the time of tagging. Minimum migration success is expressed as the proportion of fish actually detected passing through three habitats: the freshwater river, the marine inlet (Inner Dornoch, Cromarty, Chanonry, Findhorn Bay and Banff Bay) and inshore marine coastal zone (Moray Firth). Minimum migration estimates comprise simple detections of successful passage and thus do not take account of either receiver detections efficiencies or migration passage distances. There is no marine inlet at the mouth of the River Spey, thus minimum migration success through this habitat was not calculated for fish from this river; inshore marine coastal migration was also not calculated for fish from the River Deveron, as fish from this river migrated to the east and away from the main array (Array C) used to determine migration success.

Abbreviation: SD, standard deviation.

## 3 ANALYSIS OF DATA

### 3.1 Data cleaning

All detection data were cleaned to reduce spurious detections entering the analysis (Pincock, 2012). Thus detections were removed if they did not match the ID code of a deployed transmitter. Sequential detections of tags at a single receiver were removed if they were separated by less than 15 s, a set period of time less than the minimum delay between tag transmissions. Single detections were removed if there was not another detection of the same tag ID at the same receiver within 17.5 min (30 times the upper limit of the nominal delay). After these cleaning criteria were applied, the detection histories of all of the tracked fish were individually inspected to ensure that their movements were plausible. Specifically passage times were checked against known smolt swimming speeds, and sequential detections were checked using abacus plots to identify patterns of movement which did not conform to the general seaward progression that would be expected for post‐smolts. All movement patterns were deemed to conform to what was expected for post‐smolts, and all detections were retained at this stage. It is thus assumed that detections in this study are those of migrating post‐smolts.

### 3.2 Migration success

Minimum freshwater river migration success was determined as the number of fish detected on the last receiver in each river as a proportion of fish detected on the first river receiver for that river and year. Similarly, the number of fish detected on a marine inlet array as a proportion of those detected entering marine waters (i.e., on the last freshwater receiver) was used as a measure of minimum marine inlet migration success. The number of fish detected on an inshore marine coastal array expressed as a proportion of those detected leaving a marine inlet was deemed to be a measure of minimum inshore coastal marine migration success.

The RMark package in R (Laake, 2013) was used to implement capture‐mark‐recapture models [Cormack–Jolly–Seber (CJS); Cormack, 1964; Jolly, 1965; Seber, 1970] to estimate habitat‐specific migration success probability (Φ) and receiver array detection efficiency (p) (Table S2). Expected migration pathways differed among tagging rivers. For example, it was very unlikely a post‐smolt originating in the River Deveron and migrating towards known marine feeding grounds would be detected by a freshwater receiver in a river towards the west of the study area. Therefore, to calculate spatially explicit migration successes and array‐specific detection efficiencies, separate models were constructed for fish with different expected migration pathways (Table S3). Both River Oykel and River Shin origin fish were included in one model (Model 1 – Oykel/Shin) because these fish left freshwater past the same freshwater receiver (Kyle of Sutherland FW; Figure 1). Although River Cassley origin fish also exited at the same location, fish from this group were excluded from the model because they were only tagged in 1 year.

Migration success probability and receiver detection efficiency parameters were each modelled as a function of two covariates: migration success probability (Φ) was predicted from the habitat (marine inlet or coastal marine zone) through which fish migrated and year, and receiver detection efficiency (p) was predicted from receiver array identity and year (Table S1). The likely pathways for fish from the rivers Spey and Deveron had only one zone and one estimable receiver array detection efficiency, so these models only included year as a covariate (Table S3). Inestimable parameters were fixed to ensure the correct count of estimable parameters was supplied to the model. Φ was fixed to 1 for the freshwater river passage. At this point, all fish successfully entered the study; p was fixed to 1 for the final receiver array because when both Φ and p vary across space, only their product can be estimated for the final zone and final receiver array. To account for distance travelled during migration, migration success (Φ) was expressed as migration success rate per km of fish movement (i.e., Φ = 0.9 represents a 90% probability of migrating 1 km). The minimum distance by water between each pair of arrays in the study area was used as an approximation of smolt/post‐smolt movement distance. This was calculated using R with the actel package (Baktoft, 2021); the distances Matrix function was based on a raster of the Moray Firth with 40 m pixels and a transition layer with 16 movement directions resulting in an estimated precision of ±40 m.

Models were exported to the MARK package in R (Laake, 2013) for goodness‐of‐fit testing. C‐hat (an estimate of the true overdispersion parameter, c) was calculated for each pathway with sufficient data. There were insufficient data to estimate these parameters for the Conon, Spey and Deveron pathways. C‐hat was estimated by three resampling‐based methods (observed deviance divided by mean bootstrap deviance, observed C‐hat divided by mean bootstrap C‐hat and median C‐hat), then the mean of these estimates was taken as the final C‐hat estimate; 1000 bootstrap resamples were performed per bootstrap C‐hat estimate; 1200 resamples (number of intermediate points = 10 and number of replicates at each point = 100) were conducted for each median C‐hat estimate. If mean C‐hat exceeded 1, there was evidence of overdispersion, and the Akaike's information criterion (AIC) value for model‐quality assessment was converted to Quasi‐AIC (QAIC) (Howe et al., 2019).

An information theoretic approach was used to assess which candidate model best explained the data. A set of candidate models was constructed for each pathway. Each set comprised intercept models and all nested combinations of the global model's explanatory variables (Table S3). Candidate models were ranked by their information criterion score [corrected AIC (AICc) or QAICc]. There was no clear best candidate model for any of the five pathway models (top model information criterion weights ranged from 0.138 to 0.695 with a median of 0.336), so Φ and p parameters (Table S4) (Johnson and Omland, 2004) were averaged across each candidate model set using the RMark model. average function (Laake, 2013). A variance covariance matrix was calculated, and estimates from models with nonpositive beta variances were included in averaging. Averaging was restricted to those models that fitted the data reasonably well (δ AICc or δ QAICc ≤13.044; Bolker et al., 2009, Grueber et al., 2011).

### 3.3 Migration timing

Fish arrival date at all receiver arrays in the study area (Figure 1; Table S1) was analysed with a linear mixed effects model fitted by maximum likelihood using the R package lme4 (Bates et al., 2015). For each array at which a tag was detected, the arrival date was the day of year of its first detection at that array. There were 1128 arrival dates for 432 tags at 15 receiver arrays (including the freshwater receivers immediately prior to marine water entry) in 2019 and 901 arrival dates for 434 tags at 12 receiver arrays in 2021. The most complex model included an interaction between the fixed effects of year and tag river, a random intercept effect for array and a random intercept effect for individual. Random effects were included because arrays nearer the shore were likely to have earlier detection dates than those offshore and because individuals that left the river earlier would also be expected to arrive at the offshore receivers earlier. Year was a categorical variable with two levels (2019 and 2021). River was a seven‐level categorical variable (Conon, Deveron, Findhorn, Ness, Oykel, Shin, Spey). The River Cassley was not included in the model because no fish from this river were tagged in 2019. The significance of the most complex model's fixed effects was assessed with likelihood ratio tests (LRTs) with a significance threshold of α = 0.05. The null hypothesis tested was that marine migration timing does not vary among rivers or years.

The best model was used to calculate 95% prediction intervals using the default arguments of merTools' predict interval function (Knowles & Frederick, 2023). Each prediction interval was unique to an individual array, river and year combination. For each river and year, the minimum lower and maximum upper 95% prediction interval limits were extracted. These limits bounded a migration window of time within which the majority of fish were predicted to transit the study area.

### 3.4 Migration speed

Speed of migration was calculated as the minimum travel distance by water divided by the time elapsed between first detections at two different receiver arrays. To determine what factors might influence the speed of migration, this variable was used as the response variable in a linear model fitted by maximum likelihood [generalized linear models (GLM) in R]. The explanatory variables were year, river and day of year on entry to the marine waters. Interactions of year with river, year with marine entry day and river with marine entry day were also included in the model. The significance of each term was assessed using LRTs with a significance threshold of α = 0.05.

### 3.5 Inshore marine migration pathway

To determine the migration route taken by post‐smolts from each river in each year, the receiver in any array that first detected the tag of a post‐smolt was defined as the passage point for that fish. To visualise the migration pathway of a post‐smolt, a single straight‐line vector, linking detections of fish as they pass between first detections on separate arrays but avoiding crossing a land boundary, was constructed for each fish individually. These pathways represent a minimum‐distance marine passage route, as location (and thus pathway) between arrays cannot be determined.

Individual receivers were numbered sequentially from the most southerly to the most northerly at each of the four largest marine arrays [part of Array C is orientated east to west (Figure 1), the receiver sequential numbering was simply applied following the curtain from east to west at this point]. To define the most common passage point and a measure of variation around this for fish from each river of origin in each year, the median and interquartile range (25% and 75% passage point frequencies), as well as the 5% and 95% passage point frequencies rounded to the nearest integer, were determined. Migration passage point probabilities were plotted using ggplot2 package in R (Wickham, 2016).

### 3.6 Factors determining post‐smolt pathways

To investigate what factors may predict how Atlantic salmon post‐smolts were passing through the inshore coastal marine zone of the central Moray Firth, the location of first detections of tags at Array C (Figure 1) were analysed with a variable dispersion beta regression model (Cribari‐Neto & Zeileis, 2010). This modelling approach was used, firstly because the location data were scaled from 0 to 1 (see below) and, secondly, because it was of interest to explore the effect of river of origin and year on both the mean passage location and the variation around that mean. Arrival locations were converted to values on an open unit interval for use as the beta‐distributed response variable, as follows. The rank order location of the receiver first detecting a migrating post‐smolt was defined as the sequential position on the receiver array numbered from 1 (the most southerly) to 127 (the most northerly). Detection location was then scaled to between 0 and 1 using the following two equations (derived from Smithson & Verkuilen, 2006):

(1) $$y^{\prime }=\left(y-a\right)/\left(b-a\right)$$

where *y* is receiver location by rank order along the array, *a* is the minimum possible *y*, and *b* is the maximum possible *y*.

(2) $$y^{\prime \prime }=\left(y^{’*}\left(n-1\right)+0.5\right)/n$$

where *n* is sample size, and y″ is the scaled receiver position.

Two parameters define the beta distribution: location (*μ*) and precision (*τ*). At Array C, *μ* represents the mean location of fish arrivals, whereas *τ* is the inverse of the spread of post‐smolts around *μ* for fish from each river/year combination. In the most complex model, each parameter was modelled as a function of three explanatory variables: year, river of fish origin and arrival day of year, as well as their pair‐wise interactions. As River Cassley smolts were only tagged in 2021, fish from this river were not included in the model. The *μ* parameter had a logit link function, whereas *τ* used a log link.

An LRT approach was used for backward model selection to assess the significance of each term in the most complex regression model. All LRTs had a significance level of α ≤0.05 for tests between models, and the significance of terms included in the final model was assessed with further LRTs. Diagnostic plots of the residuals in each model were inspected to check model fit.

To estimate the width of the pathways bounded by the passage points where 90% and 50% of fish were determined crossing Array C, quantiles were extracted from the predicted beta distributions then back transformed from the open unit interval to the rank order receiver position in the array (of 1–127). The upper and lower limits of these prediction intervals were the receivers that matched the whole number nearest the back‐transformed quantile.

The work described in this study conformed with the regulations of the UK Animals (Scientific Procedures) Act 1986 and was conducted under licence number PP0483054.

## 4 RESULTS

### 4.1 Minimum migration success

The mean number of fish tagged in each river in each year was 103.9 (range: 48–150; Table 1). Of the total 1559 Atlantic salmon smolts tagged across all rivers combined (*n* = 8 in 2021; *n* = 7 in 2019), 898 were detected at, or beyond, the last receiver in freshwater in each river (Figure 1). These fish (*n* = 431 in 2019; *n* = 467 in 2021) entered marine waters and thus entered the study. The minimum river migration success estimated from tag detections without correction for receiver efficiency and irrespective of the migration distance across all fish from all rivers and years was 58.1%, but this varied with river of origin and year. The highest minimum river migration success was 92% for smolts originating in the Shin in 2019, and the lowest 10% was for smolts from the Ness in 2019 (Table 1). Similarly, minimum marine inlet migration success ranged from 64%, for River Deveron origin fish through Banff Bay in 2021, to 89%, for River Ness origin fish through the Beauly Firth to the Chanonry array in 2019, with a mean of 77% across all rivers and years (Table 1; Figure 1). Passage through the inshore marine coastal zone was similarly variable with a mean minimum inshore coastal zone migration success of 72%. However, this ranged from 54% for Conon origin fish in 2019 to 80% for Shin origin fish in 2021 (Table 1).

### 4.2 Coastal zone migration pathways

**FIGURE 2:**
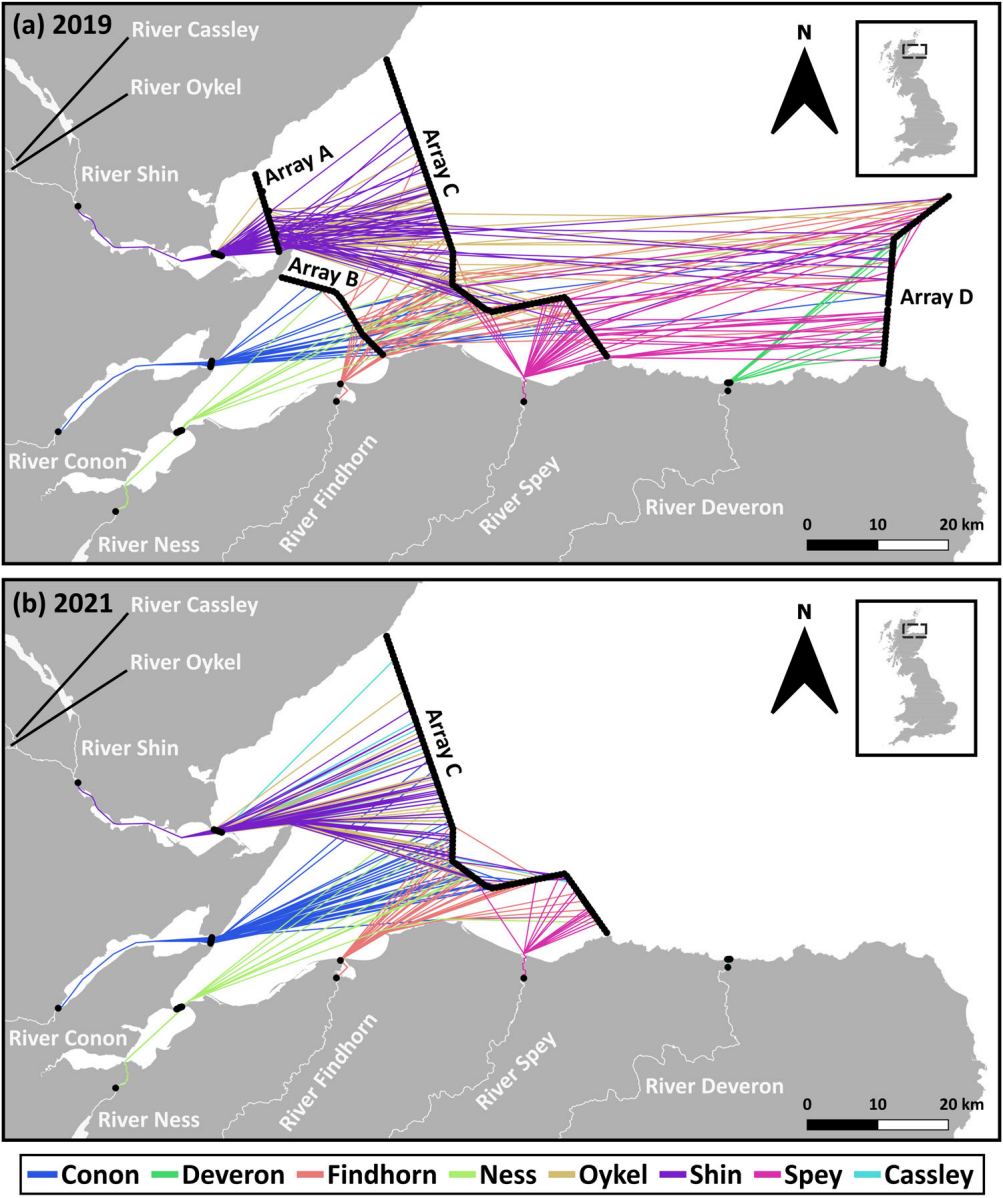
The migration pathways of Atlantic salmon post‐smolts in the Moray Firth in (a) 2019 and (b) 2021. Lines join consecutive detections of individual fish at the successive arrays (shown as black dots and lines) but were constrained to pass through only aquatic habitats. These represent a minimum migration distance pathway between detections. Pathway colours are river specific.

**FIGURE 3:**
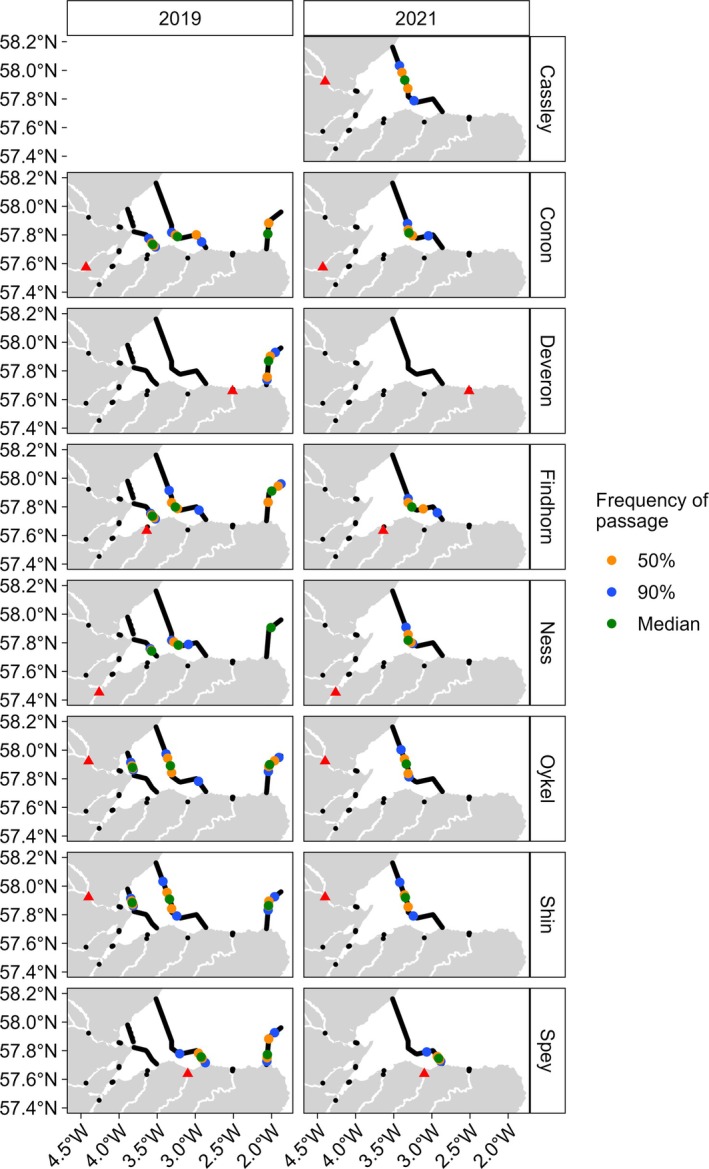
The 2019 and 2021 migration passage point boundaries. The passage of 50% of post‐smolts (orange dots; 25%–75% passage frequencies) and 90% (blue dots; 5%–95% passage frequencies) and the median passage point (green dot) from each river of origin and in each year, as they passed the four inshore coastal marine receiver arrays in the inshore marine coastal zone (in 2019, arrays A–D; in 2021, only Array C; Figure 1). No Cassley fish were tagged in 2019, and no post‐smolts from the River Deveron were detected on Array C in 2021. River of origin is marked by a red triangle in each panel.

Inshore marine coastal zone minimum migration distance pathways of each post‐smolt, from all rivers in both years of the study, are presented in Figure 2. The most‐frequent passage point for 50% and 90% of post‐smolts on each of the four inshore coastal receiver arrays (arrays A to D in 2019 and Array C only in 2021) shows a relatively consistent pattern of movement in the marine environment across rivers of origin (Figure 3).

The pathways taken by post‐smolts from all rivers were not random but skewed towards the southern part of the coastal zone study area. The straight‐line distance, south to north, to the median passage point on Array C for fish from each river ranged from 5 to 22 km north from the most southerly point on land (Table S5). This represents 8% to 48% of the south to north range of possible passage points. This is particularly clear for fish originating from those rivers that flow into the south of the study area (e.g., see the pattern for fish originating from the Spey and Findhorn rivers) (Table S5; Figure 2).

Post‐smolts originating from the River Deveron, which enters marine waters to the east of the inshore coastal receiver arrays (A–C; Figure 1), were not detected on these more western arrays. In 2019, River Deveron origin fish were detected on the southern part of Array D, which was deployed to the east of the River Deveron (Figures 1 and 2), indicating that fish from this river turn to the east after entering marine waters. Array D was not deployed in 2021, preventing an assessment of the annual consistency of migration pathway for fish from the River Deveron. Post‐smolts originating from the rivers Cassley, Oykel and Shin, which drain into the marine waters to the north‐west of the study area, were generally further north on Array C than fish from the rivers draining into the study area from the south. For example, median passage points on Array C in 2021 for fish from the rivers Cassley, Oykel and Shin were 22.4, 19.5 and 21.1 km from the most southerly land point, respectively, compared to median passage points of 9.9, 4.9 and 8.3 km from the southern shore for fish from the rivers Ness, Spey and Findhorn, respectively. However, in 2019, the slightly more northerly trajectory of fish from the rivers Oykel and Shin did not continue as fish moved eastwards, as these fish passed Array D at approximately the same location as fish originating from the rivers draining into the south of the study area (Figure 3; Table S3).

In general, the pathways used by fish passing through the coastal zone were relatively constrained in space. Thus, 50% of fish from all rivers (except those from the River Deveron) passed within 4.7 km of the mean passage point on Array C (SD = 2.07; range 2.3–6.6 km).

Variable dispersion beta regression models were used to investigate the factors that may influence both the passage point of post‐smolts at Array C (*μ*) and the variation around that mean (*τ*).

**FIGURE 4:**
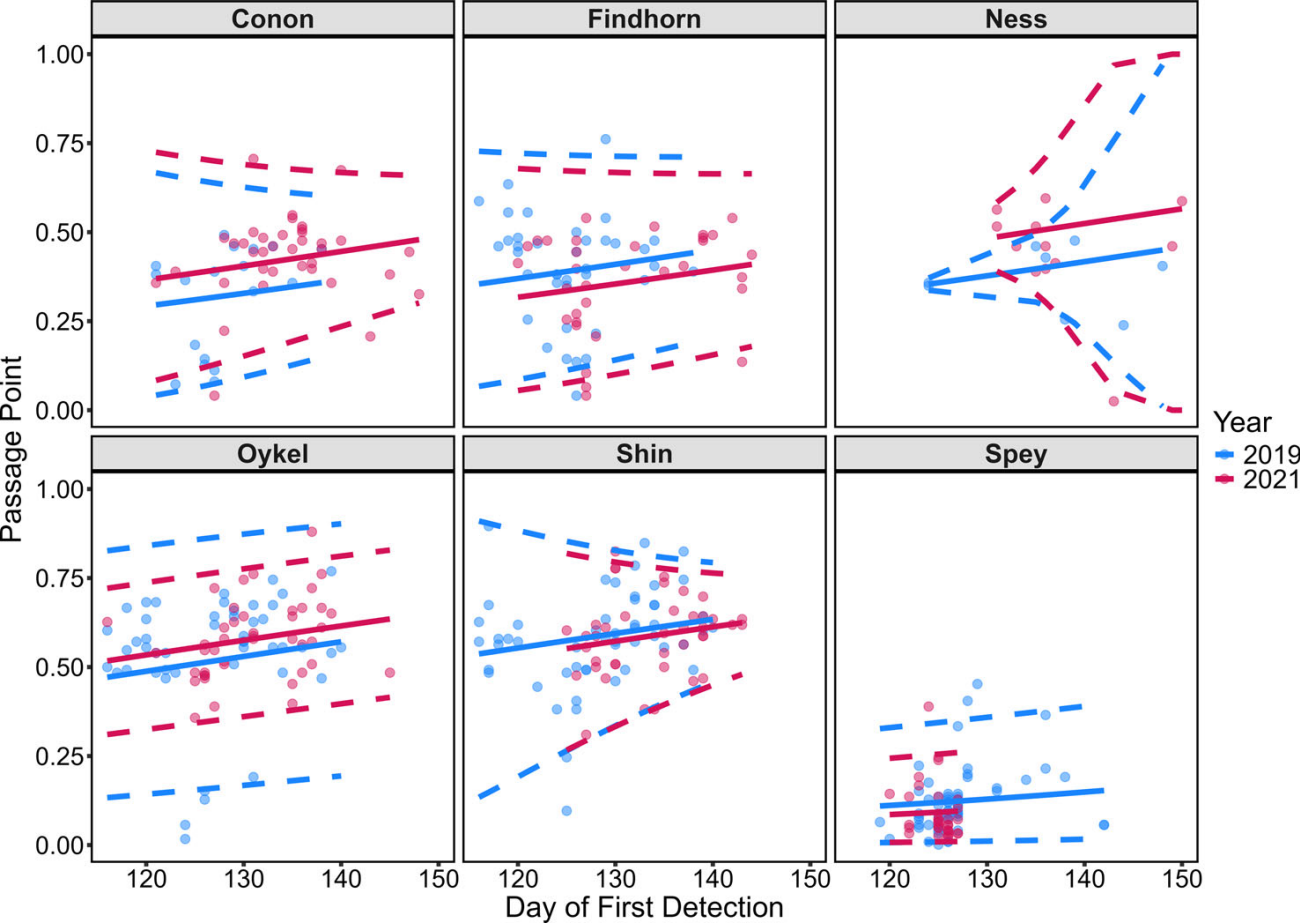
The passage point of Atlantic salmon post‐smolts on receivers from Array C (shown is the standardised passage point (see Methods). A larger number (broadly indicating passage further north) is predicted by the day of the year of first detection at the array and an interaction between year and river of origin. Model output for each of six rivers and years is shown separately. Solid lines are predicted values from the model: *μ*(arrival) ~ arrival_day of year + year*river of origin, *τ*(arrival) ~ year*river of origin + river of origin*arrival day of year. Dotted lines mark the 95% prediction intervals. Passage points are the positions of fish arrivals at Array C converted to the open unit interval (see Methods).

**FIGURE 5:**
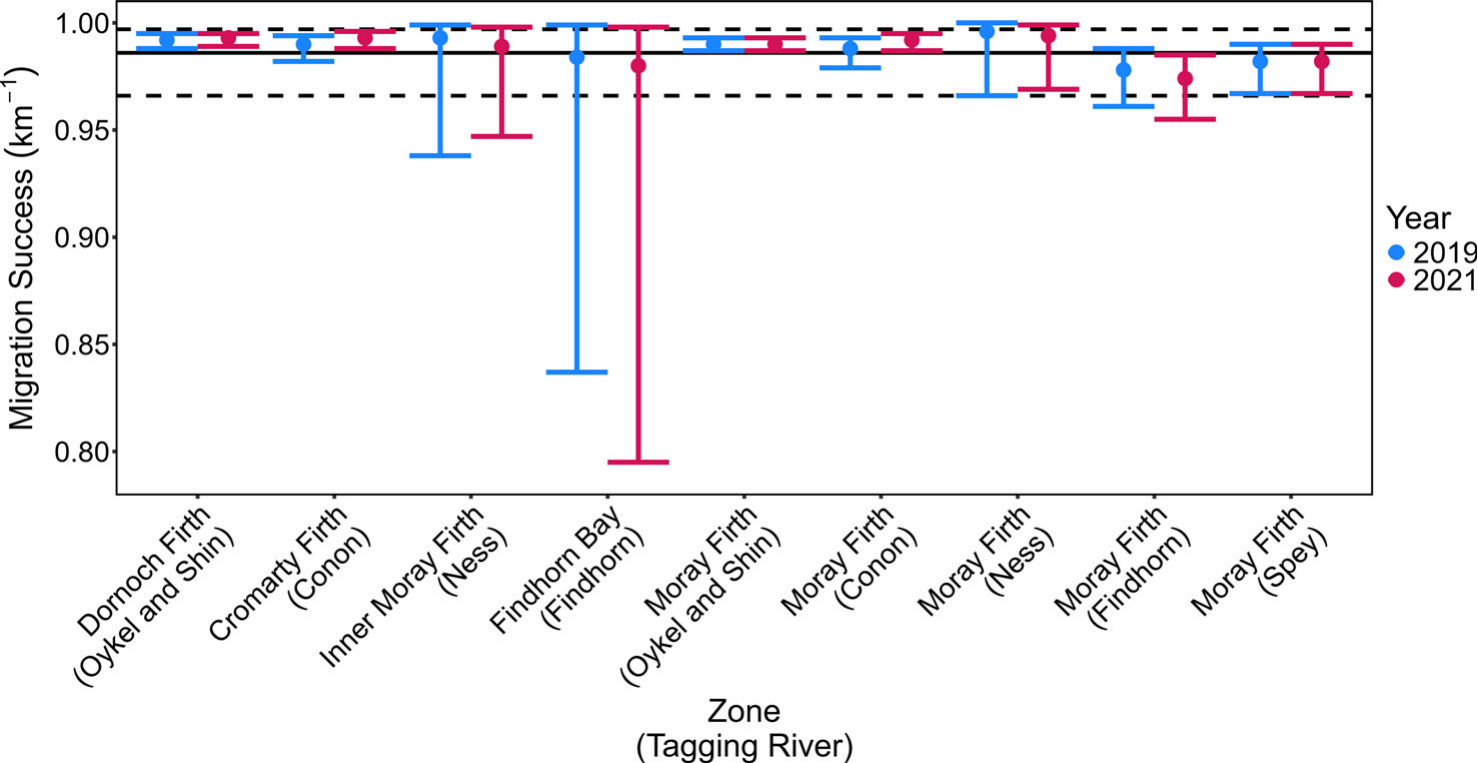
Migration success rates of Atlantic salmon post‐smolt through marine inlets and the inshore coastal marine zone of the Moray Firth for all rivers and both years of the study. (Note the migration success through Banff Bay is not presented because the low migration success estimate was potentially influenced by poor detection efficiency of receivers). For context, the horizontal lines represent the minimum (lower dotted), median (solid) and maximum (upper dotted) post‐smolt early marine migration success rates from a review of a number of studies on early marine migration (Thorstad et al., 2012).

Modelling indicated that post‐smolt passage point (*μ*) on Array C was predicted by (a) the day of the year when a fish was first detected at the array and (b) by an interaction between year and river of origin (Table S8). Post‐smolts that arrived at Array C later in the migration period tended to be detected by receivers that were further north (i.e., a larger standardised passage point; LRT: χ^2^ = 11.758, df = 1, *p* < 0.001; Figure 4). This effect was independent of river of origin or year of study, suggesting that this is a general and robust effect. The magnitude of the effect of arrival day of the year on the mean passage point (i.e., *μ*) was consistent across rivers. The effect of year on the passage point on the array (*μ*) depended on river of origin (LRT: χ^2^ = 18.066, df = 5, *p* = 0.003; Figures 4, 5). Fish from three rivers crossed Array C at more northerly receivers in 2021 compared to 2019 (non‐transformed correction coefficients for 2021: Conon = 0.332, Ness = 0.432, Oykel = 0.184). Although this effect was significant, the effect size was modest. Thus, the median array passage point moved north by 2.1, 2.5 and 1.3 km in 2021 cf. 2019 for Conon, Ness and Oykel origin fish, respectively. Fish from the other three rivers crossed Array C at more southerly receivers in 2021 compared to 2019 (non‐transformed correction coefficients for 2021: Findhorn = −0.234, Shin = −0.090, Spey = −0.292). This effect size was also modest (Table S5). These detectable river‐specific differences in migration pathways among years were small relative to differences among rivers. Fish originating from the River Spey maintained a distinctly more southerly pathway in both years compared to fish of other rivers of origin (Figures 2, 4, 5; Table S5).

The model examining the magnitude of variation around the mean passage point on Array C (*τ*) indicated that an interaction between year and river of origin and an interaction between the day of the year at first detection and river of origin predicted the magnitude of variation in the passage point (Table S8). The magnitude of the effect of the day of the year when a tag was first detected at the array on the spread of passage points around the mean passage point (*τ*) depended on river of origin (LRT: χ^2^ = 24.304, df = 5, *p* < 0.001; Figure 4).

Day of arrival at Array C weakly affected the within‐population variation around the average passage point for five of the six studied rivers [logit scale slope coefficients were between −0.003 (River Oykel fish) and 0.079 (River Shin fish)]. This effect was relatively strong for River Ness fish (logit scale slope coefficient = −0.309), but this is possibly an artefact of sample size, with only 8 and 12 Ness fish detected by the Array C in 2019 and 2021, respectively, compared to a minimum of 18 across the other tagging river‐year combinations (Table 1). These results suggest that average passage point for fish of any river of origin at Array C was fairly consistent. The lack of a significant interaction between day of year of arrival at the array and year indicates that this effect was consistent across both years in the study.

The effect of year on the variation around the mean passage point at the Array C (*τ*) depended on river of origin of the fish (LRT: χ^2^ = 13.652, df = 5, *p* = 0.018; Figures 4, 5). Fish from two of the studied rivers exhibited greater variation in passage point at Array C in 2021 compared to 2019 (logit scale correction coefficients for 2021: Findhorn = −0.0491, Ness = −1.219). Fish from the remaining four rivers showed less variation in passage point at Array C in 2021 compared to 2019 (logit scale correction coefficients for 2021: Conon = 0.089, Oykel = 1.219, Shin = 0.162, Spey = 0.428). This indicates that variation in the passage point for migrants at Array C was river dependent.

### 4.3 Marine migration success rate

The median migration success rate (Φ) across all marine inlets and the inshore coastal marine zones for all fish from all rivers of origin was 0.990 km^−1^ (minimum = 0.719 km^−1^, maximum = 0.993 km^−1^; Table S6; Figure 5). The median migration success rate across all river/year combinations through the five coastal inlets was 0.985 km^−1^ and through the coastal zone of the Moray Firth was 0.990 km^−1^.

Migration success was thus uniformly high across all component parts of the marine migration for fish from all rivers. It was also largely consistent between years, the greatest between‐year variation being for passage through the marine inlet of Banff Bay by fish originating from the River Deveron, although even here the differences in the rate of migration success between years were modest (0.896 in 2019 and 0.719 in 2021).

### 4.4 The timing of migration

**FIGURE 6:**
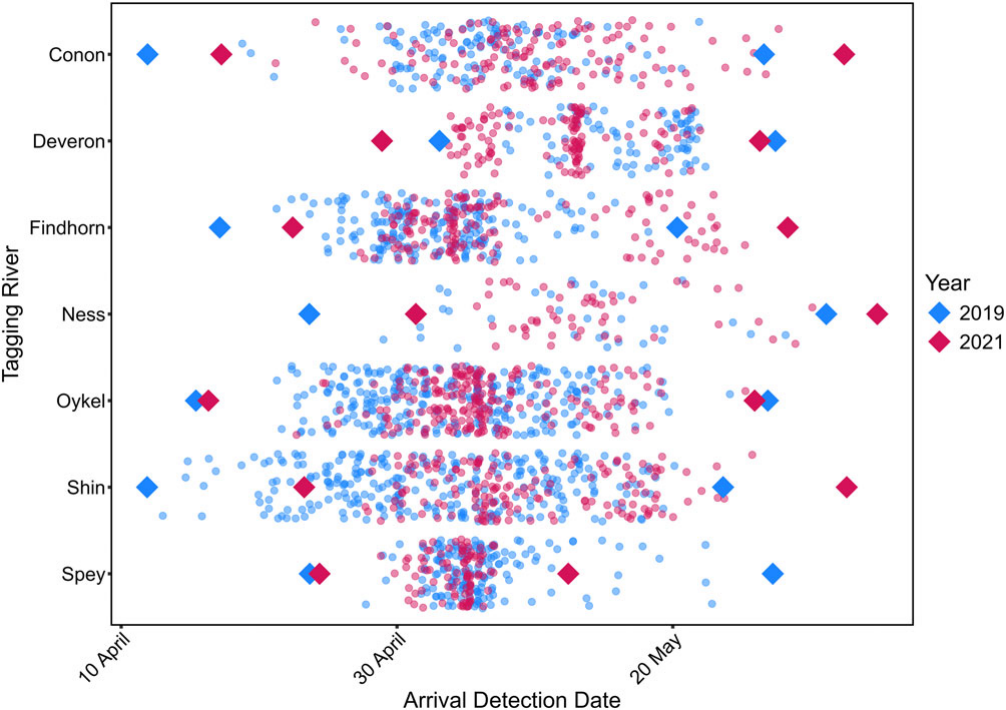
The dates of first detection of individual Atlantic salmon post‐smolts. The diamonds represent the upper and lower limits of the model‐predicted migration windows, whereas the points represent individual fish arrival detections at each receiver array.

An interaction between year and river of origin significantly predicted the date of first arrival at an array (LRT: χ^2^ = 96.30, df = 6, *p* < 0.001), indicating that the effect of year on migration timing depended on fish river of origin (Figure 6). The difference between marginal *R* ^2^ of the final model (0.25) and its conditional *R* ^2^ (0.93) indicates that the random effects (receiver array and fish ID) explained a large portion of the variation in migration timing.

The mean migration window durations across all populations and both years of the study was 36.5 days (minimum = 18 days, maximum = 45 days; Figure 6). Nonetheless between 3 and 12 May, inshore coastal marine migration occurred across all river‐year combinations. This suggests that there is considerable synchrony in the marine migration across populations in the study area.

### 4.5 Migration speed

**FIGURE 7:**
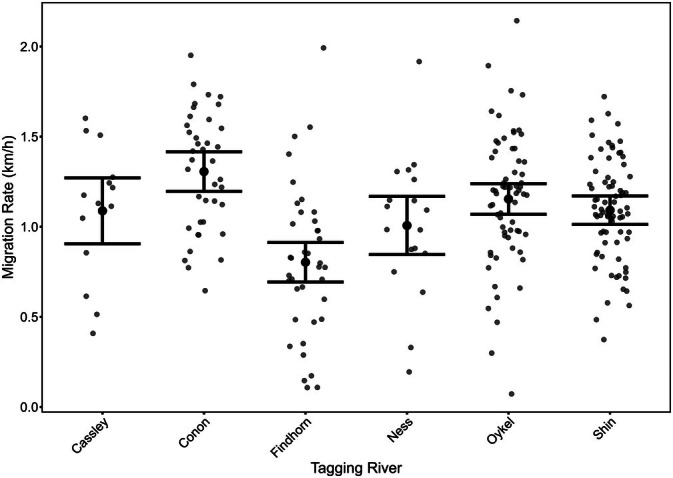
The estimated migration speeds of Atlantic salmon post‐smolt from each of six rivers through the inshore coastal marine environment of the Moray Firth. Large solid black point and error bars are model‐predicted mean migration rates for fish from each river of origin with 95% confidence interval. The small points are observed values with horizontal jitter.

Individual fish migration speeds were estimated for fish migrating through the inshore marine coastal zone of the Moray Firth from the point of exit from the marine inlet of each respective river (arrays at the Inner Dornoch, Cromarty, Chanonry and Findhorn Bay) and Array C (Figure 2). Migration rates through the Moray Firth varied among tagging rivers (LRT: χ^2^ = 42.286, df = 5, *p* < 0.001), with fish originating from the River Findhorn having the slowest migration speed and fish from the River Conon migrating the quickest (Table S7; Figure 7).

## 5 DISCUSSION

There is a general paucity of information on the migration of Atlantic salmon in inshore coastal marine waters. As is the case for many other species (Cresswell et al., 2011), the cost of migration for Atlantic salmon is high; typically less than 5% (and frequently considerably lower) of those fish making the outward migration to marine feeding areas return successfully to their natal rivers for spawning (Chaput, 2012; Chaput et al., 2019). There is also strong evidence that sea migration success in this species has declined over recent decades (Adams et al., 2022; Dadswell et al., 2021). Those empirical studies that exist on the migration success of post‐smolts on their first migration from freshwater into coastal waters largely (but not wholly) indicate that this part of the migration is associated with low success (Dieperink et al., 2001, 2002; Hedger et al., 2011; Vollset et al., 2025, 2016). In addition to all of this, the majority of pressures with potential for a negative effect on this rapidly declining species (Chaput, 2012; Nunn et al., 2023) are both disproportionately found in the inshore coastal marine costal environments and are likely expanding there (Gillson et al., 2022; Thorstad et al., 2012). There is thus a compelling need for a more complete understanding of the processes involved in Atlantic salmon migration through inshore coastal marine habitats.

In this study, we contribute to the understanding of Atlantic salmon post‐smolt migration by testing three hypotheses around key aspects of their movement through coastal waters during their first migration to sea. To do this, we tracked individual fish from eight rivers over two consecutive years, examined variation in migration patterns among individuals, between populations and, over time, within a single inshore coastal marine zone traversed by a large cohort of migrants. A possible exception to this is that of fish from the River Ness, where with only eight and 12 fish were detected on Array C, some caution must be exercised in inference drawn from post‐smolt migration for fish from this river.

### 5.1 Migration success

Migration success through both enclosed marine inlets at the mouth of the study rivers and the wider inshore coastal marine area of the Moray Firth was high. Measured crudely as the number of fish detected leaving the study area as a proportion of those entering the area, the minimum migration success takes no account of travel distance nor any variation in the detection efficiency of receivers. Marine migration rate, in contrast, does account for distance travelled during the migration. Both indices of migration success, measured across all fish in the study, were high for fish passing through marine inlets on transition from freshwater to the marine environment (overall minimum migration success 77%; median migration success rate 0.99 km^−1^) and for the wider inshore marine coastal zone (overall minimum migration success 72%; median migration success rate 0.99 km^−1^). Thus our hypothesis that migration success of post‐smolts in nearshore coastal zone will be low was not supported in this study. This finding contrasts with studies conducted elsewhere in inshore coastal marine habitats; for example, ca. 17% migration success in wild Atlantic salmon post‐smolts (Vollset et al., 2016, Vollset et al., 2025); 45% migration success within 18 days (Dieperink et al., 2001); 58% migration success in the first 15 days in marine waters (Dieperink et al., 2002). High marine migration success through both marine inlet and wider inshore coastal marine habitat was apparent across all populations (rivers of origin) and across both years, indicating that this effect was both spatially and temporally consistent, at least over the range examined in this study.

Several studies have linked low inshore coastal marine migration success of Atlantic salmon post‐smolts to high levels of predation by birds (Dieperink et al., 2001, 2002) and other fish species (gadoids) (Hedger et al., 2011). Our findings indicate that predation in the Moray Firth coastal zone, even when migrating smolts are relatively constrained in marine inlets, is markedly lower than reported elsewhere. The results from the eight rivers and 2 years of our study are more consistent with the relatively high rate of coastal marine migration success recorded by Lilly et al. (2022) in Atlantic salmon post‐smolts, as they migrated through the estuarine waters of the Clyde on the west coast of Scotland. Taken together, this suggests that migration success by Atlantic salmon post‐smolts through inshore marine coastal zone may be highly modulated by local environmental coastal conditions. What is driving the between‐coastal zone differences in migration success is not clear from the study presented here. One possibility is that either the density of predators or the vulnerability of post‐smolts may differ markedly between coastal zones. Another is that the degree of confinement of the coastal zone that smolts enter (which was relatively open in the study reported here in contrast to comparative studies) (Dieperink et al., 2001, 2002; Hedger et al., 2011; Vollset et al., 2025). Alternatively, there may be other coastal zone pressures on post‐smolt migration that differ between locations. This is clearly an area that would benefit from more research focus.

### 5.2 Coastal zone migration pathways

The pathways adopted by post‐smolts through the inshore marine coastal zone examined in this study were constructed from simple interpolation of detections of tags as they passed receiver arrays. Thus, they represent a simplified pathway representing the minimum passage route that any post‐smolt is likely to have taken.

The patterns shown by this study provide important insights into post‐smolt migration through the inshore coastal marine habitat. Post‐smolts emigrating from their natal rivers moved through the inshore coastal marine zone from west to east, towards North Sea waters, the route that would logically ultimately lead them towards the known feeding grounds in the Norwegian Sea. The pattern of pathways taken by post‐smolts through the inshore coastal marine habitat was clearly not random but was also not simply following the prevailing current (for more detail on current patterns, see Newton et al., 2021, Campbell, 2018). A few other studies have shown that Atlantic salmon post‐smolts in inshore waters are making active directional choices through active swimming (Hedger et al., 2011; Lilly et al., 2024; Newton et al., 2021; Rodger, Lilly, et al., 2025). In this study, in general, pathways were disproportionately concentrated towards the south of the study area. This indicates that in this inshore coastal marine area, fish were generally adopting similar pathways. Analysis of the between‐population variation in detected pathways is informative. It shows a relatively consistent pattern of migration pathway choice, with the between‐population median passage point on a detection array in the inshore coastal marine zone (Array C) ranging over only 5 to 22 km from the southern shore of the study area. Thus, although between‐population differences were detectable, the small effect sizes were ecologically trivial. Although detectable, the temporal effect of year on pathway adopted was inconsistent, with some populations adopting a more northerly route and others a more southerly route between years. Despite this, the actual magnitude of the effect of year was very small and, although statistically significant, we argue that it is ecologically trivial. Within‐population (among individual) variation in pathways, that is, deviation from the population mean pathway, was also relatively modest. Modelling detected a consistent effect where fish migrating later in the season chose pathways that were further north compared to earlier migrants. This effect, however, although detectable in all populations, was small and in the context of the size of the study area had only a very minor effect on the pathways exhibited. However, that this effect can be detected indicates that the pathways used by post‐smolts in the inshore coastal marine zone are influenced by extrinsic factors. Interactions between population (river of origin) and year, and population and the timing of the migration (day of the year) both had statistically detectable effects on the variation in pathways around the mean; these effects were also quantitatively small and biologically trivial. This indicates that variation in the migration pathway adopted is consistent between populations and over time, at least over the variation examined here. Thus hypothesis two, that pathways utilised by post‐smolts in the coastal zone are river specific, was not supported. This finding has implications for the management of the coastal zone examined in this study. The relatively constrained geographic nature of the use of the coastal zone provides the basis for marine planning to avoid the potential for conflict between coastal activities and post‐smolt migration.

A geographically wide‐ranging study of the migration of Atlantic salmon post‐smolts, in a single year from 25 rivers of origin, through the marine waters to the west of the UK and Ireland (Rodger, Lilly, et al., 2025), shows similarities and contrasts with the data from the study presented here. As with our study, post‐smolts from the same population (river) used broadly similar nearshore and coastal pathways. In contrast to the study presented here, however, these general pathways differed between populations. Taking previous studies together with the study presented here suggests that the extent to which there may be synchrony in pathway use between populations which originate from different rivers is likely dependent on the inshore coastal marine zone through which they are migrating. The 25 populations included in the Rodger, Lilly et al.'s (2025) study passed into inshore coastal marine zones that were relatively complex, both geographically and in their flow patterns, compared to the Moray Firth coastal zone.

### 5.3 Inshore marine migration speed

The pace at which post‐smolts progressed through the inshore coastal marine area in this study varied statistically significantly between fish from different rivers. However, although detectable, the magnitude of this effect was relatively small, with maximum variation being within 25% of the mean of all populations (1.1 km · h^−1^). Given that migration speed was determined as the minimum (straight line) travel distance between detecting receivers on successive arrays as a function of time elapsed, it is highly possible that the between‐river variation in migration speed recorded here may simply represent between‐river differences in the route taken between receivers. Newton et al. (2021) report migration speed of post‐smolts in one part of the inner Moray Firth (the Cromarty Firth) as around 1.3 km · h^−1^ during the migration in 2016 suggesting that the general pace of migration is broadly consistent across time. Elsewhere, the speed of migration in inshore waters has been shown to vary with body size and water current speed. For example, Thorstad et al. (2007) showed that larger hatchery‐reared post‐smolts migrated faster through a Norwegian fjord than smaller wild fish. Taking current speed and body size into account, Okland et al. (2006) found that post‐smolts in the inshore marine environment migrated at a mean of 1.22 body lengths · s^−1^. That migration speed is broadly consistent across studies, suggesting that post‐smolts employing longer passage routes through coastal zones where migration success is low are likely to accrue higher losses (lower survival) than those fish with shorter passage routes. That is, it may not be possible for post‐smolts to mitigate the effect of passage through a high loss rate zone by increasing passage speed.

### 5.4 The seasonal timing of migration

The timing of arrival at the inshore coastal marine array in the Moray Firth (Array C) varied with year, but that effect was influenced by the river of origin of the post‐smolts. Despite this, there was a period of 10 days in this study when fish from all rivers were migrating simultaneously past Array C. Overall, the median duration of the migration window spanned only 37 days. Thus, despite detectable between‐river and year variation in seasonality, the evidence is that the window of passage of migrants very constrained in time strongly pointing to a very high degree of migration synchronicity spatially (across populations) and over time. Thus, hypothesis three that the timing of the marine migration of post‐smolts through coastal zone will be broadly synchronous in fish among rivers and over time is supported by the study presented here. Several other studies have shown a similar pattern of migration timing. The period of Atlantic salmon smolt migration was broadly similar across four Norwegian rivers but with more subtle differences in the migration start, end and peak dates between rivers (Bjerck et al., 2021). Similarly, across a 19‐year time series from a single river, Atlantic salmon showed temporal variation in the start and end of the smolt migration but within a relatively constrained migration window of time (Harvey et al., 2020). This has implications for managing coastal zone activities that may impact post‐smolt migration, as the period of vulnerability is both relatively predictable and time constrained.

## 6 CONCLUSIONS

Atlantic salmon post‐smolt migration through both marine inlets (where fish first enter marine waters) and the more inshore coastal marine zone was typified by between‐population and temporally high migration success rates. Thus, the low migration success and high predation rates reported elsewhere were not replicated in this study. Also, contrasting with the few other studies that have been able to examine this, post‐smolts from different populations utilised broadly similar pathways through the inshore coastal marine zone. Pathway use was consistent over time in this study and a smaller study from the same area in 2016 (Newton et al., 2021). The migration period in inshore marine waters was constrained in time, and the pace of passage was consistently relatively rapid. The constrained temporal and spatial nature of coastal‐zone migration shown here provides a basis for mitigating possible pressures acting on post‐smolts during this phase of their migration. The contrast between the migration by post‐smolts identified here and those from other studies strongly suggests that the patterns of inshore marine migration are likely to be habitat specific.

## AUTHOR CONTRIBUTIONS

CEA, MN, LC, JRR: conceptualisation and planning. AJL, HMH, DT, LW, MAS, MW, RM, KM, BS, SB, CC, CD, RL, MT, KW, SR, RG, BS: field execution of the study. RF, JRR, CEA: data analysis. CEA, JRR, LC, MCL: manuscript concepts, preparation and writing. All authors involved in editing.

## FUNDING INFORMATION

This research was supported by the Missing Salmon Alliance, the Atlantic Salmon Trust and MORHL windfarm.

## Supporting information


**Table S1.** The detection locations for Atlantic salmon smolts and post‐smolts migrating from each of the eight rivers as they passed through each of the three habitats (freshwater, marine inlet and inshore coastal marine zone). Successful migration through any habitat type was determined, as detection at the freshwater detection location, the array at the exit of the marine inlet or the coastal zone array indicates successful migration of fish from freshwater into marine waters.
**Table S2.** Acoustic receiver array detection efficiency estimates (*p*) for each array, with standard error (SE), upper and lower 95% confidence interval limits (CL). Note that estimates for fish originating from the Oykel and Shin are combined for 2019 and the Oykel, Shin and Cassley for 2021 because their marine passage route was similar in this study.
**Table S3.** The six models developed to test for drivers of migration success in Atlantic salmon post‐smolts for each of six river groups in this study. Data from fish from the Rivers Oykel and Shin are combined because they have identical migration passage points through the marine inlet and inshore coastal marine zones.
**Table S4.** The rankings of models were achieved using an information theoretic approach for each of six models (See Table S3).
**Table S5.** Distances from the migration passage points to the nearest coastlines on each of the coastal zone arrays (see Figure 1) are reported for the 50% and 90% frequency boundaries, as well as for the median passage point of post‐smolts from each river of origin in 2019 and 2021. Distances to the north and south coast of the Moray Firth are presented for all inshore marine receiver arrays apart from Array A, where the distance to the nearest north and south shores was measured within the relatively constrained Dornoch Firth area (Figure 1).
**Table S6.** The model estimated migration success rate (Φ) (the proportion of successfully migrating fish per km migration distance) for fish from each river passing through five marine inlets and through the wider inshore coastal zone of the Moray Firth. Standard error (SE) and lower and upper 95% confidence limits (lCL & uCL) are presented. Note that as there was no means of determining receiver array efficiency (*p*) beyond the Array C, measures of migration success comprise both Φ and *p* effects for entries marked *.
**Table S7.** Model predicted Atlantic salmon post‐smolt migration rates (measured as a minimum direct line distance between two detection points divided by the time elapsed between detections) in the central Moray Firth.
**Table S8.** The parameters of the final variable dispersion beta regression model of migrant passage points at Array C.

## Data Availability

The data that support the findings of this study are available on request from the corresponding author. The data are not publicly available due to privacy or ethical restrictions.
