# Supplementary material for: Inshore marine coastal zone migration patterns in Atlantic salmon post‐smolts emigrating from eight rivers in north‐east Scotland
Source: J Fish Biol. 2025 Nov 20;108(2):718–33. doi: 10.1111/jfb.70280 (PMC13052459; doi:10.1111/jfb.70280)
Supplement: Supplementary file 1 — Table S1. The detection locations for Atlantic salmon smolts and post‐smolts migrating from each of the eight rivers as they passed through each of the three habitats (freshwater, marine inlet and inshore coastal marine zone). Successful migration through any habitat type was determined, as detection at the freshwater detection location, the array at the exit of the marine inlet or the coastal zone array indicates successful migration of fish from freshwater into marine waters. Table S2. Acoustic receiver array detection efficiency estimates (p) for each array, with standard error (SE), upper and lower 95% confidence interval limits (CL). Note that estimates for fish originating from the Oykel and Shin are combined for 2019 and the Oykel, Shin and Cassley for 2021 because their marine passage route was similar in this study. Table S3. The six models developed to test for drivers of migration success in Atlantic salmon post‐smolts for each of six river groups in this study. Data from fish from the Rivers Oykel and Shin are combined because they have identical migration passage points through the marine inlet and inshore coastal marine zones. Table S4. The rankings of models were achieved using an information theoretic approach for each of six models (See Table S3). Table S5. Distances from the migration passage points to the nearest coastlines on each of the coastal zone arrays (see Figure 1) are reported for the 50% and 90% frequency boundaries, as well as for the median passage point of post‐smolts from each river of origin in 2019 and 2021. Distances to the north and south coast of the Moray Firth are presented for all inshore marine receiver arrays apart from Array A, where the distance to the nearest north and south shores was measured within the relatively constrained Dornoch Firth area (Figure 1). Table S6. The model estimated migration success rate (Φ) (the proportion of successfully migrating fish per km migration distance) for fish from each river passing t [file JFB-108-718-s001.docx]

**Supplementary material**

**Supplementary Table S1.** *The detection locations for Atlantic salmon smolts and post-smolts migrating from each of the eight rivers as they passed through each of the three habitats (freshwater, marine inlet and inshore coastal marine zone). Successful migration through any habitat type was determined as detection at the freshwater detection location, the array at the exit of the marine inlet or the coastal zone array. indicates successful migration of fish from freshwater into marine waters.*

| **River of origin** | **Freshwater detection location** | **Marine Inlet** | **Marine Inlet Array** | **Coastal Zone Array** |
| --- | --- | --- | --- | --- |
| Shin | Kyle of Sutherland | Dornoch Firth | Inner Dornoch Array | Array C |
| Cassley | Kyle of Sutherland | Dornoch Firth | Inner Dornoch Array | Array C |
| Oykel | Kyle of Sutherland | Dornoch Firth | Inner Dornoch Array | Array C |
| Conon | Lower Conon | Cromarty Firth | Cromarty Array | Array C |
| Ness | Lower Ness | Beauly Firth | Chanonry Array | Array C |
| Findhorn | Lower Findhorn | Findhorn Bay | Findhorn Bay Array | Array C |
| Spey | Lower Spey | None | None | Array C |
| Deveron | Lower Deveron | Banff Bay | Banff Bay Array | None |

**Supplementary Table S2**. Acoustic receiver array detection efficiency estimates (p) for each array, with standard error (SE), upper and lower 95% confidence interval limits (CL). Note estimates for fish originating from the Oykel and Shin are combined for 2019 and the Oykel, Shin and Cassley for 2021 because their marine passage route was similar in this study.

| **River** | **Year** | **Array** | ***p* estimate** | **SE** | **Lower CL** | **Upper CL** |
| --- | --- | --- | --- | --- | --- | --- |
| Oykel and Shin | 2019 | Kyle FW | 1 | 0 | 0 | 1 |
| Oykel/Shin/Cassley | 2021 | Kyle FW | 1 | 0 | 0 | 1 |
| Oykel and Shin | 2019 | Inner Dornoch | 0.87 | 0.03 | 0.81 | 0.91 |
| Oykel/Shin/Cassley | 2021 | Inner Dornoch | 0.95 | 0.02 | 0.90 | 0.97 |
| Oykel and Shin | 2019 | Array A | 0.70 | 0.04 | 0.61 | 0.78 |
| Oykel/Shin/Cassley | 2021 | Array A | 0.86 | 0.03 | 0.78 | 0.91 |
| Conon | 2019 | Conon FW | 1.00 | 0 | 0 | 1.00 |
| Conon | 2021 | Conon FW | 1.00 | 0 | 0 | 1.00 |
| Conon | 2019 | Cromarty | 0.94 | 0.06 | 0.63 | 0.99 |
| Conon | 2021 | Cromarty | 0.60 | 0.08 | 0.44 | 0.74 |
| Ness | 2019 | Ness FW | 0.96 | 0.06 | 0.50 | 1.00 |
| Ness | 2021 | Ness FW | 0.91 | 0.09 | 0.53 | 0.99 |
| Ness | 2019 | Chanonry | 0.95 | 0.09 | 0.37 | 1.00 |
| Ness | 2021 | Chanonry | 0.88 | 0.13 | 0.41 | 1.00 |
| Findhorn | 2019 | Findhorn FW | 0.96 | 0.04 | 0.72 | 1.00 |
| Findhorn | 2021 | Findhorn FW | 0.90 | 0.06 | 0.72 | 0.97 |
| Findhorn | 2019 | Findhorn Bay | 0.57 | 0.10 | 0.37 | 0.75 |
| Findhorn | 2021 | Findhorn Bay | 0.55 | 0.11 | 0.35 | 0.74 |
| Spey | 2019 | Spey FW | 0.87 | 0.04 | 0.78 | 0.93 |
| Spey | 2021 | Spey FW | 0.82 | 0.06 | 0.68 | 0.90 |
| Deveron | 2019 | Deveron FW | 1.00 | 0 | 0 | 1.00 |
| Deveron | 2021 | Deveron FW | 1.00 | 0 | 0 | 1.00 |

***Table S3.*** *The six models developed to test for drivers of migration success in Atlantic salmon post-smolts for each of six river groups in this study. Data from fish from the Rivers Oykel and Shin are combined because they have identical migration passage points through the marine inlet and inshore coastal marine zones.*

| **Model** | **Global Model Structure** | **Number of Detected Fish** | | **Capture History Structure** | | | |
| --- | --- | --- | --- | --- | --- | --- | --- |
|  |  | **2019** | **2021** | **Capture Occasion 1** | **Capture Occasion 2** | **Capture Occasion 3** | **Capture Occasion 4** |
| Model 1 – Oykel/Shin | Φ(zone * year) p(array * year) | 186 | 145 | Tagging and Release | Kyle FW | Dornoch Array | Array C |
| Model 2 - Conon | Φ(zone * year) p(array * year | 46 | 63 | Tagging and Release | Conon FW | Cromarty Array | Array C |
| Model 3 - Ness | Φ(zone * year) p(array * year | 9 | 24 | Tagging and Release | Ness FW | Chanonry Array | Array C |
| Model 4 - Findhorn | Φ(zone * year) p(array * year | 65 | 79 | Tagging and Release | Findhorn FW | Findhorn Bay Array | Array C |
| Model 5 - Spey | Φ(year) p(year) | 87 | 52 | Tagging and Release | Spey FW | Array C | N/A |
| Model 6 - Deveron | Φ(year) p(year) | 38 | 73 | Tagging and Release | Deveron FW | Banff Bay Array | N/A |

***Table S4****. The rankings of models was achieved using an* information theoretic approach for each of six models (See table S3).

Model 1 – Oykel/Shin

The model table is primarily sorted by p. As the five lowest ranked models were the only models with constant p, there was strong evidence that p did vary. The top four models, which have a total weight of 0.715, all estimate p with array and year main effects. This suggests differences in detection efficiency among years were consistent across all receiver arrays. Within groups of models with weight > 0 and with the same p covariates, the highest ranked model always either estimated Φ with a main effect for zone or was a Φ intercept model. The high rank of Φ zone models suggests migration success varied more across space than time, but the high rank of Φ intercept models indicates that migration success variation was minimal.

Candidate model ranking table for Model 1 Oykel/Shin.

| **model** | **npar** | **AICc** | **DeltaAICc** | **weight** | **Deviance** |
| --- | --- | --- | --- | --- | --- |
| Φ(~zone)p(~array + year) | 5 | 861.545 | 0 | 0.307 | 0.578 |
| Φ(~1)p(~array + year) | 4 | 862.583 | 1.038 | 0.183 | 3.639 |
| Φ(~zone + year)p(~array + year) | 6 | 863.125 | 1.579 | 0.14 | 0.129 |
| Φ(~year)p(~array + year) | 5 | 864.113 | 2.568 | 0.085 | 3.146 |
| Φ(~1)p(~array * year) | 5 | 864.607 | 3.061 | 0.067 | 3.639 |
| Φ(~zone * year)p(~array + year) | 7 | 865.028 | 3.483 | 0.054 | 0 |
| Φ(~zone + year)p(~array * year) | 7 | 865.157 | 3.612 | 0.051 | 0.129 |
| Φ(~year)p(~array * year) | 6 | 866.141 | 4.596 | 0.031 | 3.146 |
| Φ(~zone)p(~array) | 4 | 866.948 | 5.403 | 0.021 | 8.004 |
| Φ(~zone * year)p(~array * year) | 8 | 867.065 | 5.52 | 0.019 | 0 |
| Φ(~1)p(~array) | 3 | 867.57 | 6.025 | 0.015 | 10.644 |
| Φ(~zone * year)p(~array) | 6 | 868.322 | 6.777 | 0.01 | 5.326 |
| Φ(~zone + year)p(~array) | 5 | 868.35 | 6.805 | 0.01 | 7.383 |
| Φ(~year)p(~array) | 4 | 868.959 | 7.414 | 0.008 | 10.014 |
| Φ(~zone)p(~year) | 4 | 958.962 | 97.416 | 0 | 100.017 |
| Φ(~1)p(~year) | 3 | 958.996 | 97.451 | 0 | 102.07 |
| Φ(~zone * year)p(~year) | 6 | 960.516 | 98.971 | 0 | 97.521 |
| Φ(~zone + year)p(~year) | 5 | 960.705 | 99.16 | 0 | 99.738 |
| Φ(~year)p(~year) | 4 | 960.752 | 99.207 | 0 | 101.807 |
| Φ(~zone)p(~1) | 3 | 961.047 | 99.502 | 0 | 104.121 |
| Φ(~1)p(~1) | 2 | 961.426 | 99.881 | 0 | 106.514 |
| Φ(~zone + year)p(~1) | 4 | 962.041 | 100.496 | 0 | 103.097 |
| Φ(~zone * year)p(~1) | 5 | 962.411 | 100.866 | 0 | 101.444 |
| Φ(~year)p(~1) | 3 | 962.46 | 100.915 | 0 | 105.535 |

Model 2 - Conon

The lowest ranked models had constant p suggesting that detection efficiency varied among arrays or years. All of the top nine models included array and year covariates for p, and main effects for these covariates were consistently ranked above interactive effects. The latter indicates a consistent effect of year on detection efficiency across receiver arrays. For models with weight > 0, and for a given set of p covariates, models which let Φ vary as a main effect of year were generally ranked top.

Candidate model ranking table for Model 2 Conon.

| **model** | **npar** | **AICc** | **DeltaAICc** | **weight** | **Deviance** |
| --- | --- | --- | --- | --- | --- |
| Φ(~year)p(~array + year) | 5 | 289.37 | 0 | 0.311 | 1.164 |
| Φ(~zone + year)p(~array + year) | 6 | 290.318 | 0.948 | 0.193 | 0.024 |
| Φ(~1)p(~array + year) | 4 | 291.178 | 1.808 | 0.126 | 5.045 |
| Φ(~year)p(~array * year) | 6 | 291.458 | 2.088 | 0.109 | 1.164 |
| Φ(~zone * year)p(~array + year) | 7 | 292.397 | 3.027 | 0.068 | 0 |
| Φ(~zone + year)p(~array * year) | 7 | 292.422 | 3.051 | 0.068 | 0.024 |
| Φ(~zone)p(~array + year) | 5 | 292.467 | 3.097 | 0.066 | 4.261 |
| Φ(~zone * year)p(~array * year) | 8 | 294.516 | 5.146 | 0.024 | 0 |
| Φ(~zone)p(~array * year) | 6 | 294.555 | 5.185 | 0.023 | 4.261 |
| Φ(~zone + year)p(~array) | 5 | 298.622 | 9.252 | 0.003 | 10.416 |
| Φ(~year)p(~array) | 4 | 298.735 | 9.365 | 0.003 | 12.602 |
| Φ(~zone * year)p(~array) | 6 | 298.935 | 9.565 | 0.003 | 8.641 |
| Φ(~1)p(~array) | 3 | 299.948 | 10.578 | 0.002 | 15.873 |
| Φ(~zone)p(~array) | 4 | 300.131 | 10.76 | 0.001 | 13.998 |
| Φ(~year)p(~year) | 4 | 326.631 | 37.26 | 0 | 40.498 |
| Φ(~zone * year)p(~year) | 6 | 327.453 | 38.083 | 0 | 37.159 |
| Φ(~zone + year)p(~year) | 5 | 328.507 | 39.136 | 0 | 40.301 |
| Φ(~1)p(~year) | 3 | 330.203 | 40.833 | 0 | 46.128 |
| Φ(~zone)p(~year) | 4 | 331.917 | 42.547 | 0 | 45.784 |
| Φ(~year)p(~1) | 3 | 334.365 | 44.995 | 0 | 50.29 |
| Φ(~1)p(~1) | 2 | 335.199 | 45.829 | 0 | 53.168 |
| Φ(~zone * year)p(~1) | 5 | 335.38 | 46.01 | 0 | 47.174 |
| Φ(~zone + year)p(~1) | 4 | 335.996 | 46.626 | 0 | 49.863 |
| Φ(~zone)p(~1) | 3 | 336.605 | 47.235 | 0 | 52.53 |

Model 3 - Ness

Intercept models were ranked highly for the River Ness. This suggests there was little variation in survival and detection efficiency among covariate levels. The sample size may have been insufficient to capture such variation; only 9 and 24 Ness fish were tracked in the study area in 2019 and 2021 respectively. All models had non-zero weights indicating that predictive power was spread across all candidate models.

Candidate model ranking table for Model 3 Ness.

| **model** | **npar** | **QAICc** | **DeltaQAICc** | **weight** | **QDeviance** | **chat** |
| --- | --- | --- | --- | --- | --- | --- |
| Φ(~1)p(~1) | 2 | 33.219 | 0 | 0.138 | 6.189 | 3.253 |
| Φ(~1)p(~year) | 3 | 33.802 | 0.583 | 0.103 | 4.626 | 3.253 |
| Φ(~zone)p(~1) | 3 | 33.94 | 0.722 | 0.097 | 4.764 | 3.253 |
| Φ(~year)p(~1) | 3 | 33.988 | 0.769 | 0.094 | 4.812 | 3.253 |
| Φ(~zone)p(~year) | 4 | 34.545 | 1.327 | 0.071 | 3.171 | 3.253 |
| Φ(~1)p(~array) | 3 | 34.653 | 1.434 | 0.068 | 5.476 | 3.253 |
| Φ(~year)p(~year) | 4 | 34.916 | 1.697 | 0.059 | 3.541 | 3.253 |
| Φ(~zone + year)p(~1) | 4 | 35.027 | 1.809 | 0.056 | 3.653 | 3.253 |
| Φ(~year)p(~array) | 4 | 35.371 | 2.152 | 0.047 | 3.996 | 3.253 |
| Φ(~1)p(~array + year) | 4 | 35.669 | 2.45 | 0.041 | 4.294 | 3.253 |
| Φ(~zone + year)p(~year) | 5 | 35.962 | 2.743 | 0.035 | 2.334 | 3.253 |
| Φ(~zone)p(~array) | 4 | 36.056 | 2.837 | 0.034 | 4.682 | 3.253 |
| Φ(~year)p(~array + year) | 5 | 36.506 | 3.288 | 0.027 | 2.879 | 3.253 |
| Φ(~zone + year)p(~array) | 5 | 36.929 | 3.71 | 0.022 | 3.302 | 3.253 |
| Φ(~zone * year)p(~1) | 5 | 36.969 | 3.75 | 0.021 | 3.341 | 3.253 |
| Φ(~zone)p(~array + year) | 5 | 37.307 | 4.088 | 0.018 | 3.679 | 3.253 |
| Φ(~1)p(~array * year) | 5 | 37.922 | 4.703 | 0.013 | 4.294 | 3.253 |
| Φ(~zone * year)p(~year) | 6 | 37.943 | 4.724 | 0.013 | 2.006 | 3.253 |
| Φ(~zone + year)p(~array + year) | 6 | 38.252 | 5.033 | 0.011 | 2.315 | 3.253 |
| Φ(~year)p(~array * year) | 6 | 38.816 | 5.597 | 0.008 | 2.879 | 3.253 |
| Φ(~zone * year)p(~array) | 6 | 38.859 | 5.64 | 0.008 | 2.922 | 3.253 |
| Φ(~zone)p(~array * year) | 6 | 39.616 | 6.397 | 0.006 | 3.679 | 3.253 |
| Φ(~zone * year)p(~array + year) | 7 | 40.147 | 6.928 | 0.004 | 1.842 | 3.253 |
| Φ(~zone + year)p(~array * year) | 7 | 40.62 | 7.401 | 0.003 | 2.315 | 3.253 |
| Φ(~zone * year)p(~array * year) | 8 | 42.575 | 9.356 | 0.001 | 1.842 | 3.253 |

Model 4 - Findhorn

There was little evidence that migration success varied among zones; Φ intercept and Φ year models were consistently ranked above those that included zone as a Φ covariate. The high rank of Φ intercept models suggests migration success was fairly constant. All models which did not include array as a p covariate had a weight of 0. This indicates there was variation in detection efficiency among arrays. After holding Φ covariates constant, models with an interactive effect of array and year for p were consistently ranked above those that only included main effects. This indicates there were array-specific differences in detection efficiency among years.

Candidate model ranking table for Model 4 Findhorn.

| **model** | **npar** | **QAICc** | **DeltaQAICc** | **weight** | **QDeviance** | **chat** |
| --- | --- | --- | --- | --- | --- | --- |
| Φ(~1)p(~array * year) | 5 | 170.063 | 0 | 0.179 | 5.374 | 2.873 |
| Φ(~1)p(~array) | 3 | 170.353 | 0.29 | 0.155 | 9.768 | 2.873 |
| Φ(~year)p(~array * year) | 6 | 170.892 | 0.829 | 0.118 | 4.134 | 2.873 |
| Φ(~year)p(~array) | 4 | 171.161 | 1.098 | 0.103 | 8.53 | 2.873 |
| Φ(~1)p(~array + year) | 4 | 171.719 | 1.656 | 0.078 | 9.088 | 2.873 |
| Φ(~zone)p(~array * year) | 6 | 171.922 | 1.859 | 0.071 | 5.164 | 2.873 |
| Φ(~zone)p(~array) | 4 | 172.195 | 2.133 | 0.062 | 9.565 | 2.873 |
| Φ(~year)p(~array + year) | 5 | 172.583 | 2.52 | 0.051 | 7.894 | 2.873 |
| Φ(~zone + year)p(~array * year) | 7 | 172.798 | 2.735 | 0.046 | 3.958 | 2.873 |
| Φ(~zone + year)p(~array) | 5 | 173.043 | 2.98 | 0.04 | 8.355 | 2.873 |
| Φ(~zone)p(~array + year) | 5 | 173.439 | 3.376 | 0.033 | 8.75 | 2.873 |
| Φ(~zone + year)p(~array + year) | 6 | 174.322 | 4.259 | 0.021 | 7.563 | 2.873 |
| Φ(~zone * year)p(~array * year) | 8 | 174.539 | 4.476 | 0.019 | 3.605 | 2.873 |
| Φ(~zone * year)p(~array) | 6 | 174.834 | 4.771 | 0.016 | 8.076 | 2.873 |
| Φ(~zone * year)p(~array + year) | 7 | 176.398 | 6.335 | 0.008 | 7.559 | 2.873 |
| Φ(~zone)p(~1) | 3 | 185.353 | 15.29 | 0 | 24.768 | 2.873 |
| Φ(~zone + year)p(~1) | 4 | 185.879 | 15.816 | 0 | 23.249 | 2.873 |
| Φ(~zone)p(~year) | 4 | 186.822 | 16.759 | 0 | 24.191 | 2.873 |
| Φ(~1)p(~1) | 2 | 187.134 | 17.071 | 0 | 28.584 | 2.873 |
| Φ(~year)p(~1) | 3 | 187.72 | 17.657 | 0 | 27.136 | 2.873 |
| Φ(~zone + year)p(~year) | 5 | 187.849 | 17.786 | 0 | 23.161 | 2.873 |
| Φ(~zone * year)p(~1) | 5 | 187.896 | 17.833 | 0 | 23.207 | 2.873 |
| Φ(~1)p(~year) | 3 | 188.093 | 18.03 | 0 | 27.509 | 2.873 |
| Φ(~year)p(~year) | 4 | 189.436 | 19.373 | 0 | 26.805 | 2.873 |
| Φ(~zone * year)p(~year) | 6 | 189.874 | 19.811 | 0 | 23.115 | 2.873 |

Model 5 – Spey

The two models with constant Φ make up 0.734 of the candidate model set’s total weight. This suggests that Moray Firth zone (Figure 2) migration success was consistent among years for Spey fish. Although p year models were ranked above p intercept models with the same Φ covariate, p covariate specification had little effect on model weight compared with Φ covariate specification. Therefore, there is evidence that Spey FW receiver detection efficiency varied among years, but the choice of Φ covariate was the more important determinant of model performance.

Candidate model ranking table for Model 5 Spey.

| **model** | **npar** | **AICc** | **DeltaAICc** | **weight** | **Deviance** |
| --- | --- | --- | --- | --- | --- |
| Φ(~1)p(~year) | 3 | 302.254 | 0 | 0.39 | 20.395 |
| Φ(~1)p(~1) | 2 | 302.503 | 0.249 | 0.344 | 22.692 |
| Φ(~year)p(~year) | 4 | 304.297 | 2.043 | 0.14 | 20.374 |
| Φ(~year)p(~1) | 3 | 304.532 | 2.278 | 0.125 | 22.673 |

Model 6 – Deveron

The two Φ year models constitute 0.941 of the candidate model set’s total weight; this is strong evidence that Banff bay migration success varied among years. However, as Banff Bay was the terminal zone for this model, detection efficiency would confound estimated migration success. Therefore, among-years migration success variation would be a function of both differential migration success and differential Banff Bay receiver array detection efficiency. P intercept models had more than double the weight or year varying p models with the same Φ covariates. This indicates that Deveron FW receiver detection efficiency was constant among years.

Candidate model ranking table for Model 6 Deveron.

| **model** | **npar** | **AICc** | **DeltaAICc** | **weight** | **Deviance** |
| --- | --- | --- | --- | --- | --- |
| Φ(~year)p(~1) | 3 | 131.9 | 0 | 0.695 | 0 |
| Φ(~year)p(~year) | 4 | 133.974 | 2.074 | 0.246 | 0 |
| Φ(~1)p(~1) | 2 | 137.445 | 5.546 | 0.043 | 7.601 |
| Φ(~1)p(~year) | 3 | 139.501 | 7.601 | 0.016 | 7.601 |

**Table S5**. The distances from the migration passage points to the nearest coastlines on each of the coastal zone arrays (see Figure 1 and Table 2) for the 50% and 90% frequency boundaries and the median passage point for post-smolts from each river of origin in 2019 and 2021. Distances to the north and south coast of the Moray Firth are presented for all inshore marine receiver arrays apart from Array A where the distance to the nearest north and south shores was measured within the relatively constrained Dornoch Firth area (Figure 1).

| **River of origin** | **Receiver array** | **Year** | **Distance to nearest coast (dist. to north coast km; dist. to south coast km)** | | | | |
| --- | --- | --- | --- | --- | --- | --- | --- |
|  |  |  | **90% upper** | **50% upper** | **median** | **50% lower** | **90% lower** |
| Cassley | C | 2021 | 15.09; 33.9 | 20.41; 28.45 | 25.66; 22.43 | 27.04; 15.96 | 33.03; 7.52 |
| Conon | D | 2019 | 83.11; 20.36 | 83.11; 20.36 | 88.22; 11.98 | 88.22; 11.98 | 88.22; 11.98 |
| Conon | B | 2019 | 12.83; 10.31 | 16.69; 6.21 | 18.05; 4.83 | 19.43; 3.43 | 20.79; 2.04 |
| Conon | C | 2019 | 27.86; 9.87 | 31.09; 8.31 | 33.03; 7.52 | 47.03; 12.11 | 52.24; 5.61 |
| Conon | C | 2021 | 26.81; 16.61 | 27.48; 11.9 | 28.51; 9.62 | 31.74; 8 | 43.81; 12.97 |
| Deveron | D | 2019 | 83.76; 25.83 | 82.56; 22.64 | 83.92; 18.96 | 91.56; 6.39 | 92.9; 4.28 |
| Findhorn | D | 2019 | 85.44; 30.03 | 84.56; 27.87 | 82.88; 23.5 | 86.47; 14.76 | 86.47; 14.76 |
| Findhorn | B | 2019 | 15.39; 7.56 | 16.69; 6.21 | 17.69; 5.2 | 19.43; 3.43 | 20.79; 2.04 |
| Findhorn | C | 2019 | 25.88; 20.46 | 27.58; 11.21 | 31.09; 8.31 | 33.03; 7.52 | 49.59; 8.89 |
| Findhorn | C | 2021 | 27.28; 14.32 | 27.58; 11.21 | 31.09; 8.31 | 39.99; 11.35 | 51.37; 6.6 |
| Ness | D | 2019 | NA | NA | 82.72; 23.07 | 82.72; 23.07 | 82.72; 23.07 |
| Ness | B | 2019 | 14.74; 8.25 | 16.04; 6.88 | 16.69; 6.21 | 16.69; 6.21 | 16.69; 6.21 |
| Ness | C | 2019 | 27.86; 9.87 | 29.79; 9.02 | 33.68; 7.37 | 33.97; 7.33 | 41.26; 12.22 |
| Ness | C | 2021 | 25.99; 19.81 | 27.28; 14.32 | 27.86; 9.87 | 30.44; 8.66 | 31.74; 8 |
| Oykel | D | 2019 | 84.78; 28.4 | 83.56; 25.35 | 82.42; 22.25 | 82.87; 20.73 | 85.18; 16.86 |
| Oykel | A | 2019 | 8.6; 6.19 | 9.57; 3.44 | 10.29; 2.06 | 10.7; 1.38 | 11.46; 0.23 |
| Oykel | C | 2019 | 21.7; 27.1 | 24.98; 23.75 | 26.43; 17.88 | 27.4; 12.59 | 49.08; 9.56 |
| Oykel | C | 2021 | 18.47; 30.49 | 25.62; 23.09 | 26.13; 19.15 | 27.48; 11.9 | 28.51; 9.62 |
| Shin | D | 2019 | 83.56; 25.35 | 82.28; 21.76 | 84.34; 18.25 | 84.52; 17.95 | 86.47; 14.76 |
| Shin | A | 2019 | 8.6; 6.19 | 9.29; 4.12 | 9.92; 2.75 | 10.7; 1.38 | 11.46; 0.23 |
| Shin | C | 2019 | 15.09; 33.9 | 23.7; 25.07 | 25.99; 19.81 | 27.4; 12.59 | 32.39; 7.73 |
| Shin | C | 2021 | 15.77; 33.21 | 25.62; 23.09 | 25.79; 21.12 | 27.29; 13.98 | 32.39; 7.73 |
| Spey | D | 2019 | 83.56; 25.35 | 83.11; 20.36 | 90.47; 8.16 | 92.01; 5.68 | 93.78; 2.92 |
| Spey | C | 2019 | 34.92; 7.44 | 49.08; 9.56 | 51.7; 6.25 | 52.77; 4.94 | 56.01; 1.18 |
| Spey | C | 2021 | 42.52; 12.93 | 51.37; 6.6 | 52.77; 4.94 | 53.83; 3.6 | 54.91; 2.31 |

***Table S*6*.*** *The model estimated migration success rate (Φ) (the proportion of successfully migrating fish per km migration distance) for fish from each river passing through five marine inlets and through the wider inshore coastal zone of the Moray Firth. Standard error (SE) and lower and upper 95% confidence limits (lCL & uCL)are presented. Note that as there was no means of determining receiver array efficiency (p) beyond the Array C, measures of migration success comprise both Φ and p effects for entries marked *.*

| **Migration zone** | **River of origin** | **Year** | **Migration success estimate (Φ) (km^-1^)** | **SE** | ***l*CL** | ***u*CL** |
| --- | --- | --- | --- | --- | --- | --- |
| **Marine Inlets** |  |  |  |  |  |  |
| Inner Dornoch Firth | Oykel and Shin | 2019 | 0.992 | 0.002 | 0.988 | 0.995 |
| Inner Dornoch Firth | Oykel and Shin | 2021 | 0.993 | 0.002 | 0.989 | 0.995 |
| Cromarty Firth | Conon | 2019 | 0.90 | 0.003 | 0.982 | 0.995 |
| Cromarty Firth | Conon | 2021 | 0.99 | 0.003 | 0.984 | 0.998 |
| Inner Moray Firth | Ness | 2019 | 0.99 | 0.008 | 0.938 | 0.999 |
| Inner Moray Firth | Ness | 2021 | 0.99 | 0.009 | 0.947 | 0.998 |
| Findhorn Bay | Findhorn | 2019 | 0.98 | 0.02 | 0.837 | 0.999 |
| Findhorn Bay | Findhorn | 2021 | 0.98 | 0.026 | 0.795 | 0.998 |
| Banff Bay | Deveron | 2019 | 0.896* | 0.05 | 0.751 | 0.961 |
| Banff Bay | Deveron | 2021 | 0.719* | 0.048 | 0.616 | 0.804 |
| **Inshore Coastal Zones** |  |  |  |  |  |  |
| Moray Firth | Oykel & Shin | 2019 | 0.99* | 0.001 | 0.987 | 0.993 |
| Moray Firth | Oykel & Shin | 2021 | 0.99* | 0.001 | 0.987 | 0.993 |
| Moray Firth | Conon | 2019 | 0.99* | 0.035 | 0.271 | 1 |
| Moray Firth | Conon | 2021 | 0.99* | 0.019 | 0.546 | 1 |
| Moray Firth | Ness | 2019 | 0.99* | 0.004 | 0.966 | 1 |
| Moray Firth | Ness | 2021 | 0.99* | 0.005 | 0.969 | 0.999 |
| Moray Firth | Findhorn | 2019 | 0.978* | 0.007 | 0.961 | 0.988 |
| Moray Firth | Findhorn | 2021 | 0.974* | 0.007 | 0.955 | 0.985 |
| Moray Firth | Spey | 2019 | 0.982* | 0.005 | 0.967 | 0.99 |
| Moray Firth | Spey | 2021 | 0.982* | 0.006 | 0.967 | 0.99 |

**Table S7**. Model predicted Atlantic salmon post-smolt migration rates (measured as minimum direct line distance between two detection point divided by the time elapsed between detections) in the central Moray Firth.

| **River of origin** | **Model predicted migration rate (km.h^-1^)** | **Standard error** |
| --- | --- | --- |
| Ness | 1.01 | 0.082 |
| Findhorn | 0.80 | 0.056 |
| Oykel | 1.15 | 0.043 |
| Shin | 1.09 | 0.040 |
| Conon | 1.31 | 0.056 |
| Cassley | 1.09 | 0.093 |

**Table S8**: The parameters of the final variable dispersion beta regression model of migrant passage points at Array C.

| **Component** | **Term** | **Estimate** | **Std. Error** | **Z.Value** | **P.Value** |
| --- | --- | --- | --- | --- | --- |
| Mean (μ) | (Intercept) | -2.887 | 0.603 | -4.791 | < 0.001 |
| Mean (μ) | year2021 | 0.332 | 0.179 | 1.85 | 0.064 |
| Mean (μ) | tag_riverfindhorn | 0.351 | 0.189 | 1.855 | 0.064 |
| Mean (μ) | tag_riverness | 0.215 | 0.159 | 1.353 | 0.18 |
| Mean (μ) | tag_riveroykel | 0.836 | 0.19 | 4.411 | < 0.001 |
| Mean (μ) | tag_rivershin | 1.099 | 0.172 | 6.374 | < 0.001 |
| Mean (μ) | tag_riverspey | -1.193 | 0.192 | -6.223 | < 0.001 |
| Mean (μ) | arrival_doy | 0.017 | 0.005 | 3.698 | < 0.001 |
| Mean (μ) | year2021:tag_riverfindhorn | -0.566 | 0.234 | -2.419 | 0.016 |
| Mean (μ) | year2021:tag_riverness | 0.1 | 0.204 | 0.489 | 0.62 |
| Mean (μ) | year2021:tag_riveroykel | -0.148 | 0.218 | -0.679 | 0.5 |
| Mean (μ) | year2021:tag_rivershin | -0.422 | 0.203 | -2.085 | 0.037 |
| Mean (μ) | year2021:tag_riverspey | -0.624 | 0.252 | -2.476 | 0.013 |
| Precision (τ) | (Intercept) | -4.4 | 4.315 | -1.02 | 0.31 |
| Precision (τ) | year2021 | 0.088 | 0.447 | 0.198 | 0.84 |
| Precision (τ) | tag_riverfindhorn | 2.693 | 5.4 | 0.499 | 0.62 |
| Precision (τ) | tag_riverness | 50.701 | 6.978 | 7.266 | < 0.001 |
| Precision (τ) | tag_riveroykel | 6.632 | 5.191 | 1.278 | 0.2 |
| Precision (τ) | tag_rivershin | -3.195 | 5.227 | -0.611 | 0.54 |
| Precision (τ) | tag_riverspey | 7.084 | 6.389 | 1.109 | 0.27 |
| Precision (τ) | arrival_doy | 0.052 | 0.034 | 1.536 | 0.12 |
| Precision (τ) | year2021:tag_riverfindhorn | -0.138 | 0.569 | -0.242 | 0.81 |
| Precision (τ) | year2021:tag_riverness | -1.308 | 0.76 | -1.722 | 0.085 |
| Precision (τ) | year2021:tag_riveroykel | 1.13 | 0.537 | 2.105 | 0.035 |
| Precision (τ) | year2021:tag_rivershin | 0.073 | 0.548 | 0.134 | 0.89 |
| Precision (τ) | year2021:tag_riverspey | 0.339 | 0.552 | 0.615 | 0.54 |
| Precision (τ) | tag_riverfindhorn:arrival_doy | -0.021 | 0.042 | -0.497 | 0.62 |
| Precision (τ) | tag_riverness:arrival_doy | -0.361 | 0.052 | -6.903 | < 0.001 |
| Precision (τ) | tag_riveroykel:arrival_doy | -0.055 | 0.041 | -1.358 | 0.17 |
| Precision (τ) | tag_rivershin:arrival_doy | 0.027 | 0.041 | 0.657 | 0.51 |
| Precision (τ) | tag_riverspey:arrival_doy | -0.053 | 0.05 | -1.063 | 0.29 |
